# shinyMBA: a novel R shiny application for quality control of the multiplex bead assay for serosurveillance studies

**DOI:** 10.1038/s41598-024-57652-4

**Published:** 2024-03-28

**Authors:** Zachary Matson, Gretchen Cooley, Nishanth Parameswaran, Ashley Simon, Bettina Bankamp, Melissa M. Coughlin

**Affiliations:** 1 Viral Vaccine Preventable Diseases Branch, Division of Viral Diseases, National Center for Immunization and Respiratory Diseases, Centers for Disease Control and Prevention, Atlanta, GA USA; 2 Division of Parasitic Diseases and Malaria, Center for Global Health, Centers for Disease Control and Prevention, Atlanta, GA USA; 3 Laboratory Branch, Coronavirus and Other Respiratory Viruses Division, National Center for Immunization and Respiratory Diseases, Centers for Disease Control and Prevention, Atlanta, GA USA

**Keywords:** Software, Applied immunology

## Abstract

The multiplex bead assay (MBA) based on Luminex xMAP technology can be used as a tool to measure seroprevalence as part of population immunity evaluations to multiple antigens in large-scale serosurveys. However, multiplexing several antigens presents challenges for quality control (QC) assessments of the data because multiple parameters must be evaluated for each antigen. MBA QC parameters include monitoring bead counts and median fluorescence intensity (MFI) for each antigen in plate wells, and performance of assay controls included on each plate. Analyzing these large datasets to identify plates failing QC standards presents challenges for many laboratories. We developed a novel R Shiny application, shinyMBA, to expedite the MBA QC processes and reduce the risk of user error. The app allows users to rapidly merge multi-plate assay outputs to evaluate bead count, MFI, and performance of assay controls using statistical process control charts for all antigen targets simultaneously. The utility of the shinyMBA application and its various outputs are demonstrated using data from 32 synthetic xPONENT files with 3 multiplex antigens and two population serosurveillance studies that evaluated 1200 and 3871 samples, respectively, for 20 multiplexed antigens. The shinyMBA open-source code is available for download and modification at https://github.com/CDCgov/shinyMBA. Incorporation of shinyMBA into Luminex serosurveillance workflows can vastly improve the speed and accuracy of QC processes.

## Introduction

Serological surveillance studies (i.e., serosurveys) are essential for assessing population immunity by detecting and quantifying antigen-specific antibodies. Data produced by these studies provide information critical for guiding immunization strategies to identify susceptible populations and measure disease burden^1–8^. The multiplex bead assay (MBA) using Luminex xMAP technology is increasingly used for serosurveys because it offers benefits including multiplexing antigens, reduced serum testing volumes, and amenability to higher throughput testing compared to other serologic assays. This technology has been used for serosurveys in many different countries to evaluate immunity to vaccine-preventable diseases, as well as seroprevalence for malaria and other neglected tropical diseases^9–13^.

MBA-based studies require ongoing quality control (QC) assessments of multiple assay criteria for each antigen target. These include, evaluation of bead counts for each antigen in individual wells, evaluation of median fluorescence intensity (MFI) patterns across plates, and performance of assay controls^14^. The multitude of analytes in the MBA results in complex, multi-dimensional data that pose a challenge to conducting QC in an efficient manner. While the MBA enables high-throughput data collection, methods for organizing and analyzing QC data are lacking. Results and associated metadata must be manually extracted from each Luminex xPONENT instrument output file into another software program before analyses can be performed. ‘Manual’ analysis using Microsoft Excel spreadsheets or similar software may be adequate for smaller scale studies; however, this method becomes inefficient, labor-intensive, and prone to user error when applied in studies analyzing thousands of samples with large numbers of assay plates, each requiring QC analysis for multiple unique antigen targets.

Here we describe the development of a novel MBA QC tool, shinyMBA, designed to expedite complex serosurvey QC processes. shinyMBA was developed using the R Shiny package which allows for development of a user-friendly interactive application without requiring coding knowledge to use the application^15^. Applications built within Shiny can perform sophisticated data analyses while allowing for a broad user group and flexibility in user defined parameters and visualizations. In addition, the underlying Shiny reactive programming model ensures that computationally heavy processes within an application are executed only when necessary. This can translate to a more seamless user experience by reducing long wait times that would incur from redundantly re-executing computationally expensive algorithms. Although born from the need to optimize serosurvey QC protocols, shinyMBA is suitable for use in Luminex MBA research and laboratory QC applications outside of this scope as well. This app improves the speed at which MBA data can be evaluated, preventing unnecessary plate failures through rapid identification of QC issues and readily merging data for further analysis.

## Materials and methods

### Data processing, flow, and user interface

**Figure 1:**
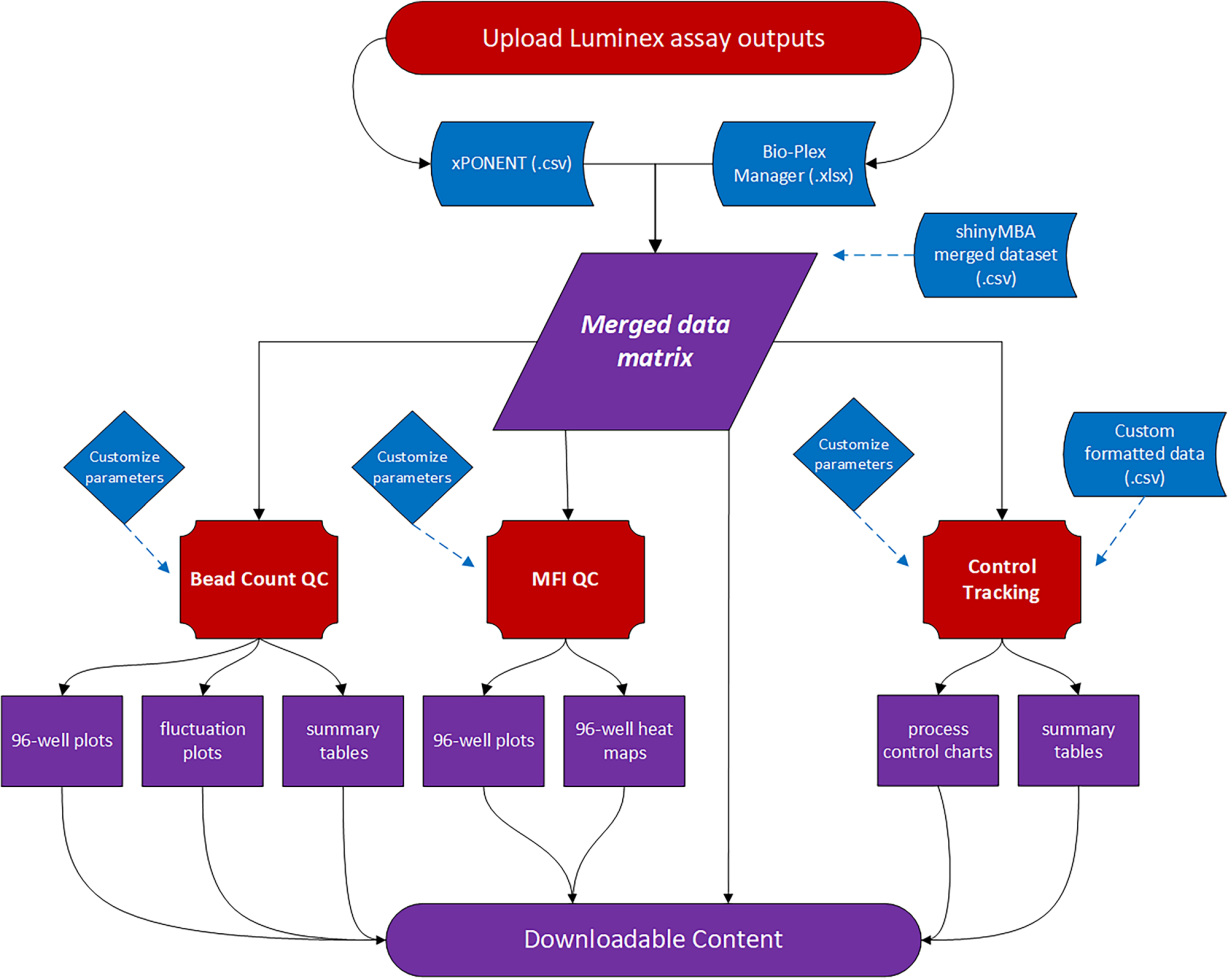
shinyMBA process flow and modular organization. The diagram provides an overview of how outputs are generated following upload of xPONENT and/or Bio-plex Manager (BPM) files. Red shapes represent hard-coded server processes, blue shapes represent inputs from the user (i.e., reactive sources), and purple shapes represent dynamic outputs (i.e., reactive endpoints). Outputs from Luminex assays are pre-processed individually and then merged into a single data table. The various plots and summary tables from each module are generated by analyzing this data table in accordance with the parameters set by the user. An exception to this is the control tracking module, which allows for a custom formatted dataset to be used instead if desired.

shinyMBA was designed with a modular interface separating the main features into four distinct modules: upload, bead count/MFI, control tracking, and downloads. The modular organization and its associated dataflow are shown schematically in Fig. 1 and described in terms of the Shiny reactive programming model.

**Figure 2:**
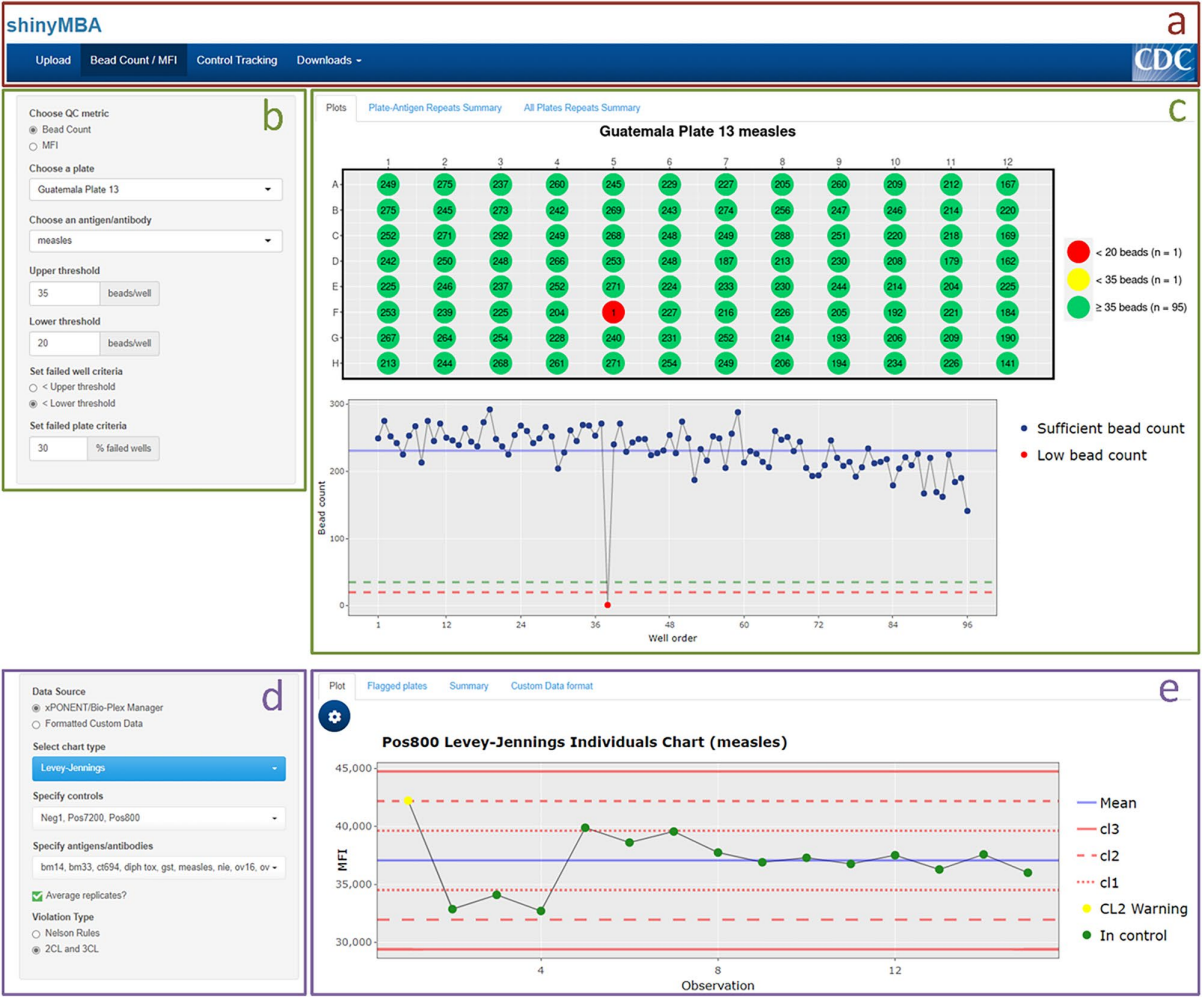
shinyMBA user interface (UI) structure. General UI layout of the Bead Count / MFI (b,c) and Control Tracking (**d**,**e**) modules. Modules are accessed using the navigation bar (**a**) at the top of the display. The Downloads module has an additional drop-down menu that navigates the types of downloadable content available. The left side of each module contains a widget panel (**b**,**d**) for customizing parameters. The right side of each module contains a dynamic active output panel (**c**,**e**) that displays plots and summary tables. Downloadable content and active output displays are updated in accordance with the parameters set in the widget panel.

Inputs were coded to function as reactive sources that instruct hard-coded server processes (i.e., expressions and observers) to update various reactive endpoints (e.g., QC plots, data tables, etc.). A single data frame was generated by merging multiple uploaded xPONENT and/or BioPlex Manager instrument output files into the Shiny app, which are pre-processed individually and then merged together. The merged data frame extracts data from the input files into the following variable columns: well location, sample ID, target antigen, MFI, net MFI, bead count, batch name, batch execution date and time, and instrument serial number. To prevent unnecessary re-executions of the computationally expensive file merging algorithm, the merged data frame was designed as a central reactive conductor from which each module would acquire its source data. An exception to this is the control tracking module, which was designed to also allow the upload of a simplified four-column .csv file to generate statistical process control charts and summary tables. The user interface (UI) was designed using the *shiny*::sidebarLayout() function to ensure a generalized, consistent template across each of the modules (Fig. 2). A top-level navigation bar was implemented to divide each module into a distinct page. Widgets, located on the left-hand side of the UI, provide an area for user input and were added using *shiny* and *shinyWidgets* UI components. These components allow users to upload files into the application, set QC parameters, and download outputs.

When applicable, interactive QC plots and data tables are displayed on the main panel of the application page. The interactive plots were generated using the *plotly* package and contain relevant metadata accessible by hovering the screen cursor over datapoints in the plot area. Configuration options to subset datapoints and display specified plot areas are present as well. This option was also designed as part of the interactive data tables using the *data.table* package which allows for basic operations such as selection of a data subset or sorting to be performed directly in the application. Additional R packages used to develop shinyMBA are described in Supplementary Table 1.

### Upload module

The upload module was designed as the portion of the application that receives instrument output files and generates the central dataset described above. The upload module was built to accept both xPONENT (.csv) and Bio-Plex Manager (BPM, .xlsx) Luminex assay files exclusively or in combination. However, as coded the BPM files must be in the multiple analyte layout and must have “FI”, “FI—Bkgd”, and “Bead Count” tabs from which shinyMBA pulls data to input into the single reactive dataframe. If an uploaded BPM file is missing any of the required attributes an alert window will appear. shinyMBA extracts data and identifies samples and controls to properly clean and merge data, requiring labelling of the input files. To ensure successful data extraction, it is advised that only original instrument output files be used. At the time of this publication, shinyMBA will only read-in output files containing single 96-well plate runs; single output files containing multiple 96-well plates or 384-well plate formats are not accepted. Merged datasets from previous shinyMBA sessions may be uploaded alongside or in lieu of instrument output files instead. When uploaded together, the central dataset is composed of instrument output files appended to the merged dataset.

### Bead count/MFI module

The Bead Count/MFI module visualizes bead count and MFI data for each uploaded plate by antigen-assigned bead region. For bead count, two plots were designed in the app: a 96-well plate plot and a bead count fluctuation plot. The plate plot was created using *ggplot2* and displays bead counts by plate well and antigen selected by the user at the widgets on the left side of the UI. User set thresholds for upper and lower bead count are identified by yellow and red colored wells, respectively. The fluctuation plot displays a line graph of bead counts per well according to the read order of the instrument which was defined in the uploaded instrument output files. Horizontal reference lines for the upper threshold, lower threshold, and the average bead counts were added to improve the visual utility of the fluctuation plot. Well location and bead count data are layered onto the points using the *plotly* R package and can be viewed by hovering the cursor over individual datapoints. Two interactive summary tables were also generated by combining the results of each plate’s bead count analysis. The first summary table utilizes *dplyr*::summarize() to calculate the proportion of failed wells for each antigen-plate combination as defined by the inputs in the UI. The second summary table performs additional calculations on the first table to generate a list and count of antigens with low bead counts for each plate.

Within this module MFI data for each plate and antigen are visualized using a 96-well plate plot structured similarly to the *ggplot2* plate plot for bead count with net MFI (MFI minus background signal) displayed for each well. Similar to the bead count widgets, the left side of the UI was designed to allow users to set a “warning” net MFI parameter. The background MFI is extracted from the Luminex instrument output file and used to identify wells at or below background MFI. These wells are automatically flagged red, while those at or below the “warning” limit are colored yellow. For plates with multiple background wells, the application was designed to utilize the well with the highest MFI to set the threshold at or below background (i.e. red wells). A second visualization option was added using *ggplot2*::geom_tile() to display the MFI heatmaps using tile plots that represent each 96-well plate. The numerical text data was added alongside a logarithmic color scale. The data to define the heatmaps is extracted from the MFI column without the background signal removed and log transformed to prevent undefined values.

### Control tracking module

 Three chart methods for process control were coded into shinyMBA: Levey-Jennings, Individuals-Moving Range (I-MR) charts, and Shewhart charts. To display these charts, MFI data were extracted from the central data frame. The display is controlled by the widgets in the UI in which antigens and controls may be selected by the user. To allow display tracking over time, the extracted MFI data were grouped by each control-antigen combination and sorted chronologically using the run date metadata extracted from each uploaded file. This input resulted in the chronological display of the observations which were plotted on the y-axis with the x-axis representing the control MFI-bkg results over time. The distance from the mean was measured in standard deviations (σ) to set the control limits using the *Rscp* package which are displayed on the process charts using dotted, dashed, and solid horizontal reference lines for 1σ, 2σ, and 3σ, respectively.

I-MR charts contain an additional plot where the chronological point-to-point MFI moving range was plotted on the y-axis alongside horizontal reference lines for the mean moving range and upper confidence limit. Point-to-point moving range was determined by calculating that absolute value of the MFI difference between two consecutive date-ordered datapoints. To define the upper confidence limit, the mean moving range was multiplied by the appropriate constant D_4_ = 3.267 as described in Montgomery et al.^17^. The method of calculating standard deviation differs between the three options for process control charts. Levey-Jennings charts use the long-term population estimate of σ whereas I-MR charts use a short-term estimate of σ based on the average moving range between two successive datapoints (*n* = 2) applying the appropriate constant *d*_2_ = 1.128 as described in Montgomery et al.^16,17^. Shewhart charts calculate control limits from a user-specified number of input files (e.g., the first 20 MBA plates run in a study) and set the control limits for the remaining study assay plates.

Controls and antigens identified in batch files were visualized in interactive process control charts using the *plotly* package and evaluated by a user-selected protocol using either the Nelson rule violations or points falling outside the 2σ or 3σ limits for the specified control-antigen pair. Nelson rule violations and points falling outside of the 2σ–3σ limits were calculated using the *Rspc* package and displayed to each control chart for reference^18^. The dataset generated from the control tracking analysis was modified into two separate interactive data tables using the *data.table* package. Using this package, the table under the “Flagged plates” tab included metadata for each displayed point in the process control chart, specifically, observation number, batch execution date, control, antigen, batch name, MFI, repeat status, and Nelson rule violation number (if applicable). A second table, found under the “Summary” tab, also generated using the *data.table* package and *dplyr*::summarize(), was developed to display descriptive summary statistics for each control and antigen combination and included the number of missing datapoints; mean, median, max, and min MFI; MFI standard deviation; 1–3σ upper and lower control limits; % of data points considered out of control; and number of each violation type.

### Downloads module

The “Downloads” module was designed as a single point from which final QC output based on user set limits may be downloaded. The downloadable output in this module was curated to enhance the visual utility of the QC plots by showing plate-wide trends for bead count and MFI or antigen-wide trends for control tracking, provide an organized summary of users’ session QC results, and furnish merged datasets suitable for easy importation into outside data analysis software such as Microsoft Excel, R, Python, SAS, or SPSS. In addition, shinyMBA was developed to use parallel processing via the *future* and *furrr* packages for plots and the *data.table* package (specifically *data.*table::fwrite()) for datasets. Types of downloadable content are described in Table 1. The downloadable plots were designed as trellised versions of the plots displayed in the “Bead Count / MFI” and “Control Tracking” modules that contain facets for each antigen across an entire plate or facets for each control across a single antigen, respectively. This visualization style was implemented to disseminate plate-wide results and comprehensive control performance more efficiently.

**Table 1:** Downloads module content.

Section	File name	Output type	Description
Raw and summary data output	Raw_data.csv	Data set	Merged dataset of all uploaded xPONENT and/or BPM files. Rows represent observations per well per antigen per batch. Variables include well location, sample ID, antigen, MFI, bead count, bead count thresholds, date and time, instrument serial number, batch name, and file name
	Raw_data_wide.csv	Data set	Merged dataset of all uploaded xPONENTand/or BPM files. Rows represent observations per well per batch with several MFI and bead count columns for each antigen. Main variables include well location, sample ID, MFI, bead count, bead count thresholds, date and time, instrument serial number, batch name, and output file name
	Summary_results.xlsx	Summary table	Four tables that summarize the following bead count repeat results: % of wells below the selected upper threshold, % of wells below the selected lower threshold, % of failed wells for each antigen within a plate, and total number of failed antigens per plate
Bead QC plots	Bead-QC-plots.zip	Plots	High resolution bead count fluctuation plots for each plate faceted by antigen
Bead count plate plots	Bead-QC-plate-plots.zip	Plots	High resolution bead count whole plate plots for each plate faceted by antigen
MFI plate plots	MFI-QC-plate-plots.zip	Plots	High resolution MFI whole plate plots for each plate faceted by antigen
MFI plate heat maps	MFI-Heat-Maps.zip	Plots	High resolution MFI heat maps for each plate faceted by antigen
Control tracking plots	Control_tracking_plots.zip	Plots	High resolution control tracking plots for selected control plot evaluation for each specified antigen faceted by control
Control tracking summary results	2cl_3cl_repeat_summary.csv	Data set	Dataset used to generate control tracking results for selected method for 2CL-3CL violations
	2cl_3cl_summary.csv	Summary table	Summary statistics table for control tracking results for selected method using 2CL-3CL violations
	Nelson_repeat_summary.csv	Data set	Dataset used to generate control tracking results for selected method for Nelson rules violations
	Nelson_summary.csv	summary table	Summary statistics table for control tracking results for selected method using Nelson rules violations

### Demonstration datasets

The utility of the shinyMBA application for MBA QC was demonstrated here using data from two Pan American Health Organization (PAHO)-supported serosurveillance studies that included 1200 and 3871 dried blood spots tested for 20 multiplexed antigens in Guatemala and Guyana, respectively^19^. The antigen panel is described in Supplementary Table 2 and included vaccine-preventable diseases, neglected tropical diseases, soil-transmitted helminths, and malaria. For Guatemala, xPONENT output files were uploaded into shinyMBA from 15 study plates. For Guyana, BPM output files were uploaded from 48 study plates.

In addition to the case study demonstration datasets, a 32 plate 3-panel synthetic xPONENT dataset was used to illustrate row-wise and column-wise patterns of MFI at or below background levels and validate data analysis methods used in the control tracking module. The synthetic data was developed to demonstrate shinyMBA capabilities and sourced from 32 deidentified xPONENT files from Luminex MBAs performed in the Viral Vaccine Preventable Diseases Branch at the Centers for Disease Control and Prevention (Atlanta, Georgia, USA). Each file contained results for a 96-well 1–3 antigen panel MBA with a 10-step threefold standard curve ran in singlet and 4 controls, a background well, and 38 samples ran in duplicate. The files titled “plate 10” and “plate 25” were randomly selected to have bead count values manually altered to bias the overall bead counts lower. The files titled “plate 1” and “plate 2” were randomly selected to have MFI values manually altered to demonstrate row-wise and column-wise patterns of MFI at or below background levels. The files titled “plate 7”, “plate 11”, “plate 14”, and “plate 29” were randomly selected to have control MFI values manually altered to bias control tracking charts towards flagging additional datapoints as out of range. These synthetic xPONENT files are available on the shinyMBA GitHub website (https://github.com/CDCgov/shinyMBA).

### Validation of bead count and control tracking analyses

The shinyMBA bead count flagging analysis was validated using the following synthetic xPONENT files: “plate 6”, “plate 10”, “plate 14”, “plate 17”, and plate “25”. Bead count data for each antigen was manually copied and pasted into Microsoft Excel bead count fluctuation charts with ≥ 35 beads/well and < 20 beads/well set as the upper and lower flagging thresholds, respectively. The same xPONENT files were then uploaded into shinyMBA and bead count flagging analysis was performed using identical flagging thresholds. The shinyMBA bead count flagging results and fluctuation plots were downloaded and compared to the Excel results to determine the accuracy of datapoint flagging.

Validation of control tracking data analysis with the shinyMBA app was performed using all 32 synthetic xPONENT datasets. Process control charts from data entered into Excel were compared to outputs of shinyMBA. MFI for each antigen-control combination was manually copied and pasted into Microsoft Excel charts formulated to perform I-MR control tracking with 2CL and 3CL datapoint flagging. Alternatively, the synthetic datasets were uploaded into shinyMBA, and I-MR control tracking with 2CL and 3CL violation flagging was performed. The shinyMBA control tracking results were downloaded and compared to the Excel charts to assess the accuracy of datapoint flagging, confidence limit calculations, and missing datapoint handling.

### Analysis of demonstration datasets

Evaluations of bead counts were conducted to identify the numbers of wells that fail to reach a statistically reliable minimum bead count and low bead count percentages across plates were compiled for each study. For the purposes of demonstrating the utility of the shinyMBA app, analysis of bead counts was performed using shinyMBA’s default parameters; wells with bead counts < 20 beads/well were considered failed wells and plates with ≥ 30% failed wells for a given antigen were considered failed plates. Additionally, plate-wide patterns of MFI using heatmaps were depicted.

In the datasets from Guyana and Guatemala, three controls were evaluated in both studies. A pool of positive sera diluted in assay buffer to 1:800 (pos800) and 1:7200 (pos7200) served as high and low positive signal controls, respectively in addition to a negative serum control. Control performance across all antigens for the dataset from Guyana was demonstrated using Levey-Jennings, I-MR and Shewhart charts that tracked control MFI readings throughout each study and identified deviations from the acceptable range. Control tracking using the Shewhart chart method was not performed for the dataset from Guatemala due to an insufficient number of plates (a minimum of 20 plates is recommended for this method). Duplicate control wells were averaged to yield a single control datapoint for each plate by selecting the “Average replicates?” option in the UI widget. For the purposes of demonstrating the utility of the shinyMBA app, datapoints detected at or outside the 3σ range were considered out of control. The first 20 chronological plates in the dataset from Guyana were used as reference points to determine the Shewhart chart control ranges.

## Results

Evaluation of bead count by well is an important QC parameter for Luminex assays and a dedicated module in shinyMBA. Low bead counts can result from pipetting errors, bead aggregation, or instrument clogging within the probe or fluidics system. Sufficiently high bead counts per well for each antigen ensure that statistically valid results are being obtained. Per the performance recommendation from Luminex, ≥ 35 beads/well is desired, however, based on reproducibility studies in our laboratory the default setting in the app was set to 20, but can be adjusted by the user (unpublished data).

To determine the bead count flagging capabilities of the app, the bead count specific data analysis methods in the Bead Count/MFI module were validated using 5 of the 32 synthetic xPONENT datasets (i.e., plates) to compare shinyMBA’s results to a bead count flagging Excel chart. The 5 files used for validation were intentionally chosen because they contained a variety of high and low bead count results. Bead count flagging comparisons between the two methods resulted in a 100% agreement (Supplementary Fig. 1). The bead count fluctuation plots generated from shinyMBA and Excel are shown in Supplementary Figs. 2–11.

**Figure 3:**
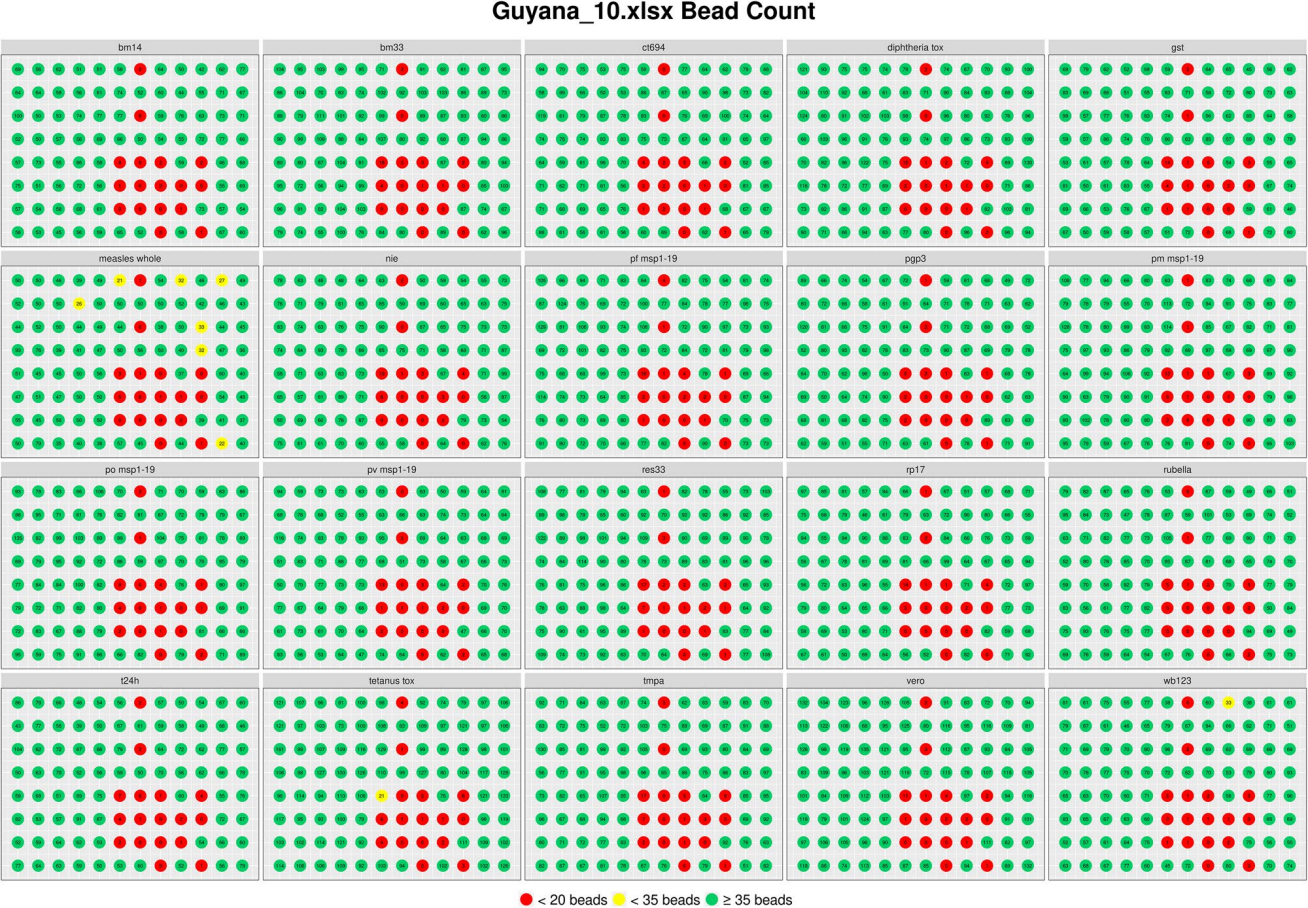
Example of a bead count plot for whole plate, faceted by antigen. Plot for plate 10 of the Guyana dataset was generated from shinyMBA bead count/MFI module using the application’s default flagging criteria, yellow (< 35 beads/per well), and red (< 20 beads/well). The figure was downloaded using the downloads module. Numeric text in the figure describes each well’s bead count.

**Figure 4:**
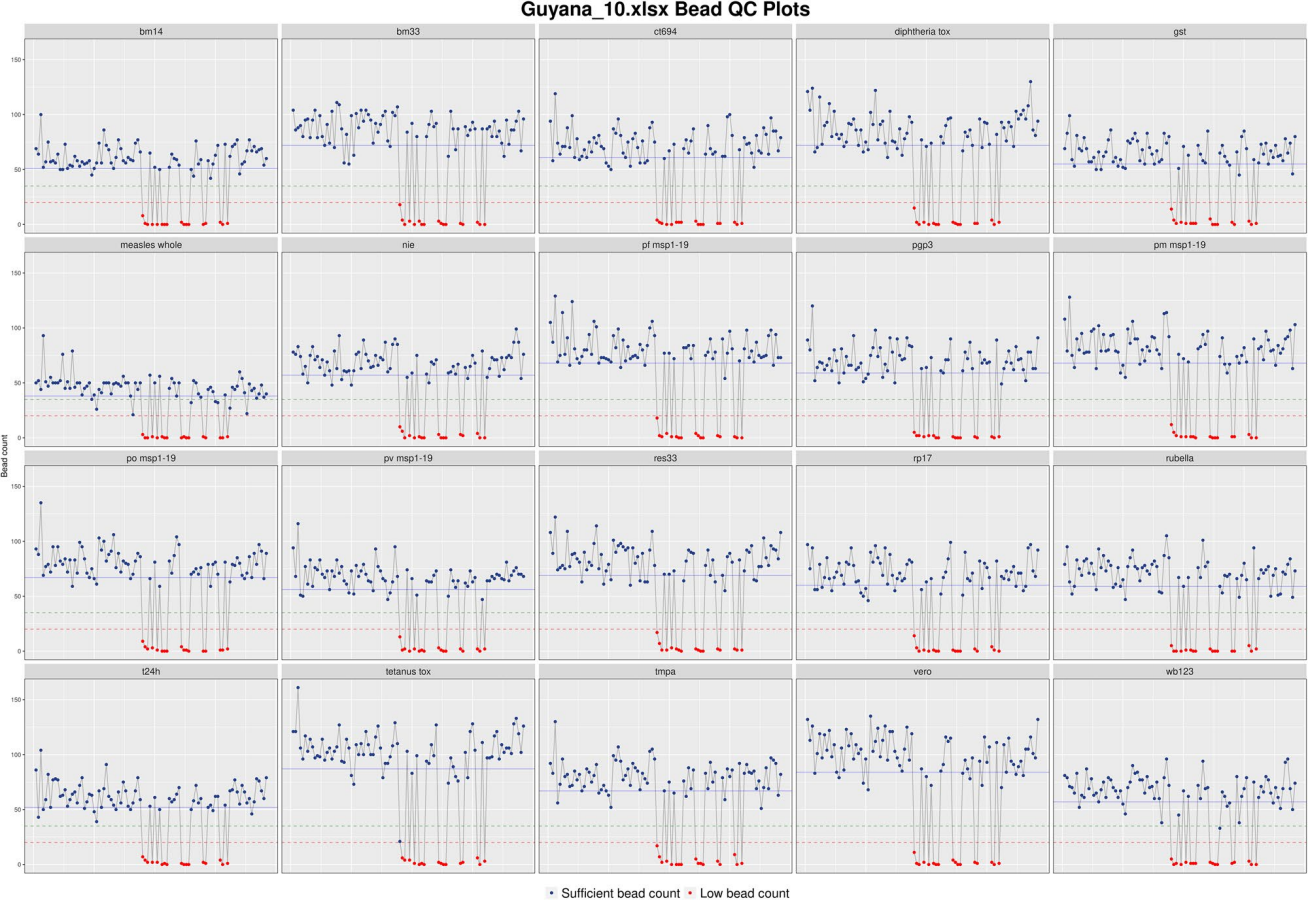
Example of a bead count fluctuation plot. Plate 10 from the Guyana study was downloaded from shinyMBA. The bead count upper threshold was set at 35 beads/well (green reference line) and the lower threshold was set at 20 beads/well (red reference line). The blue reference line indicates the mean bead count. The x axis represents individual plate wells by instrument read order. Samples with bead counts under the lower threshold were visualized as red points on the plot. The figure was downloaded using the downloads module.

Plots for bead count across whole plates were generated to identify notable patterns of low bead count. An example using the 10th plate from the Guyana case study is shown in Fig. 3. Wells with bead counts below 20 were highlighted red and wells below 35 were highlighted in yellow. The bead fluctuation plots graphed the bead count in individual wells by instrument read order and demonstrated the low bead count trend starting in the middle of the run (Fig. 4). Interactive summary tables are also generated from user input information in this module that identify plates with low bead counts based on user input and calculate overall percentage of wells in each plate that do not meet the acceptance criteria.

**Figure 5:**
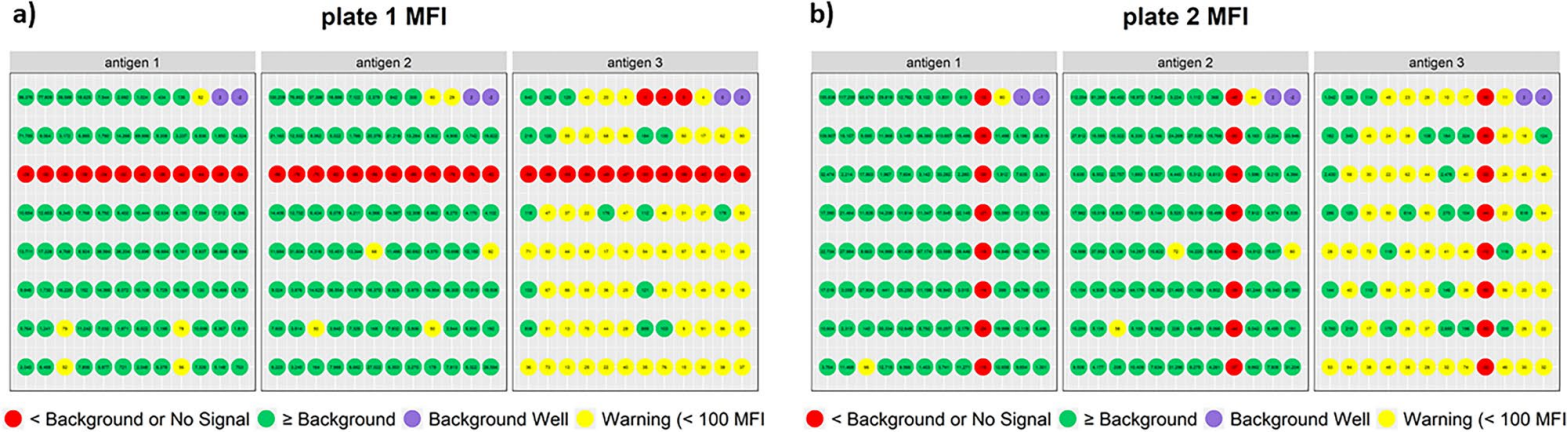
Examples of MFI whole plate plots. Plots were generated from modified sample data output files plate 1 (**a**) and plate 2 (**b**) hosted on the shinyMBA GitHub website. Red wells indicate samples with MFI readings at or below the background of the individual plate. Yellow wells indicate samples with MFI readings below the warning threshold of 100 MFI. Numeric text in the figure describes each well’s net MFI. The figures were downloaded using the downloads module.

The MFI whole plate plot flags wells either red, for wells below background, or yellow, based on user input “warning” MFI, to allow users to quickly scan for patterns across antigens and individual plates. This allows the detection of user errors such as incorrect addition of primary and secondary antibodies or detection reagents. Such errors can be visualized in the MFI whole plate plots as column- or row-wise patterns of MFI at or below background levels across multiple antigens (Fig. 5). Row-wise and column-wise pipetting errors were demonstrated in Fig. 5 using two synthetic datasets as no such pattern was present in either case study. Because the MFI component of the Bead Count / MFI module does not have a method of determining plate failures, no validation was performed.

**Figure 6:**
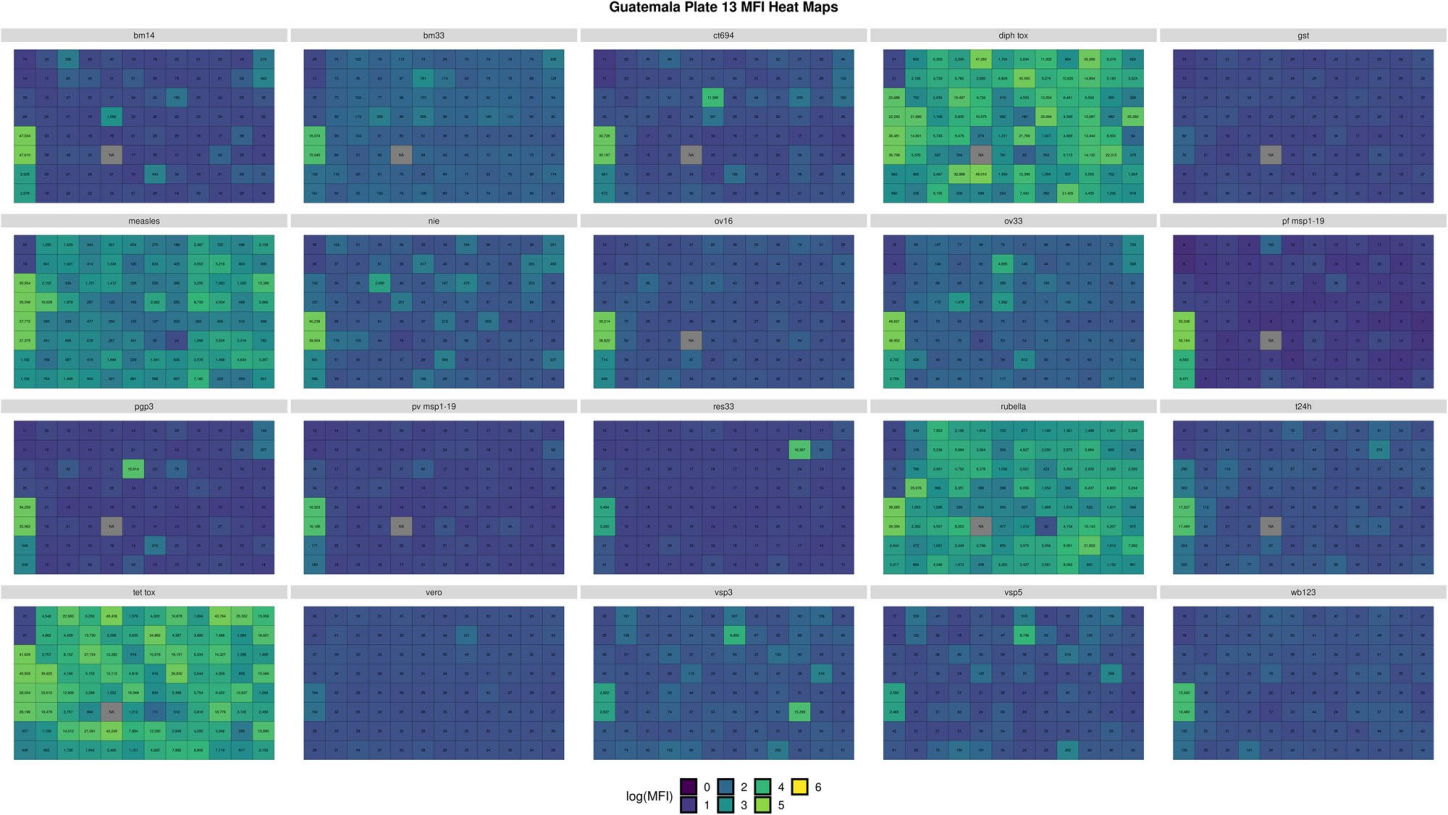
Example of a MFI heat map for plate 13 from the Guatemala study. Numeric text in the figure describes each well’s MFI. The heatmap color scale used log-transformed MFI values without background subtraction with gray tiles labeled “NA” representing missing data. The figure was downloaded using the downloads module.

The logarithmic color scale MFI heatmaps visualized the pattern of signal as described in the methods bead count/MFI module. As shown for the representative case study plate, this output additionally provided a quick qualitative assessment of which antigens produced noticeable high or low signals in each plate. Of the 20 antigens tested, diphtheria toxoid, measles, rubella, and tetanus toxoid antigens generated the highest MFI signals across the plate (Fig. 6).

To analyze the process control performance of the app, the data analysis methods in the Control Tracking module were validated using a set of 32 synthetic xPONENT datasets (i.e. plates) with 4 controls and 3 antigens to compare shinyMBA’s I-MR results to an I-MR Excel chart. Of the 32 plates, 29 contained data for “antigen 1”, 19 contained for data for “antigen 2”, and all 32 plates contained data for “antigen 3”. Datapoint flagging comparisons between both methods resulted in a 100% agreement (Supplementary Fig. 12). Additional comparison results for confidence limit calculations, missing datapoint handling, and control chart generation are shown in Supplementary Table 3 and Supplementary Figs. 13–24.

**Figure 7:**
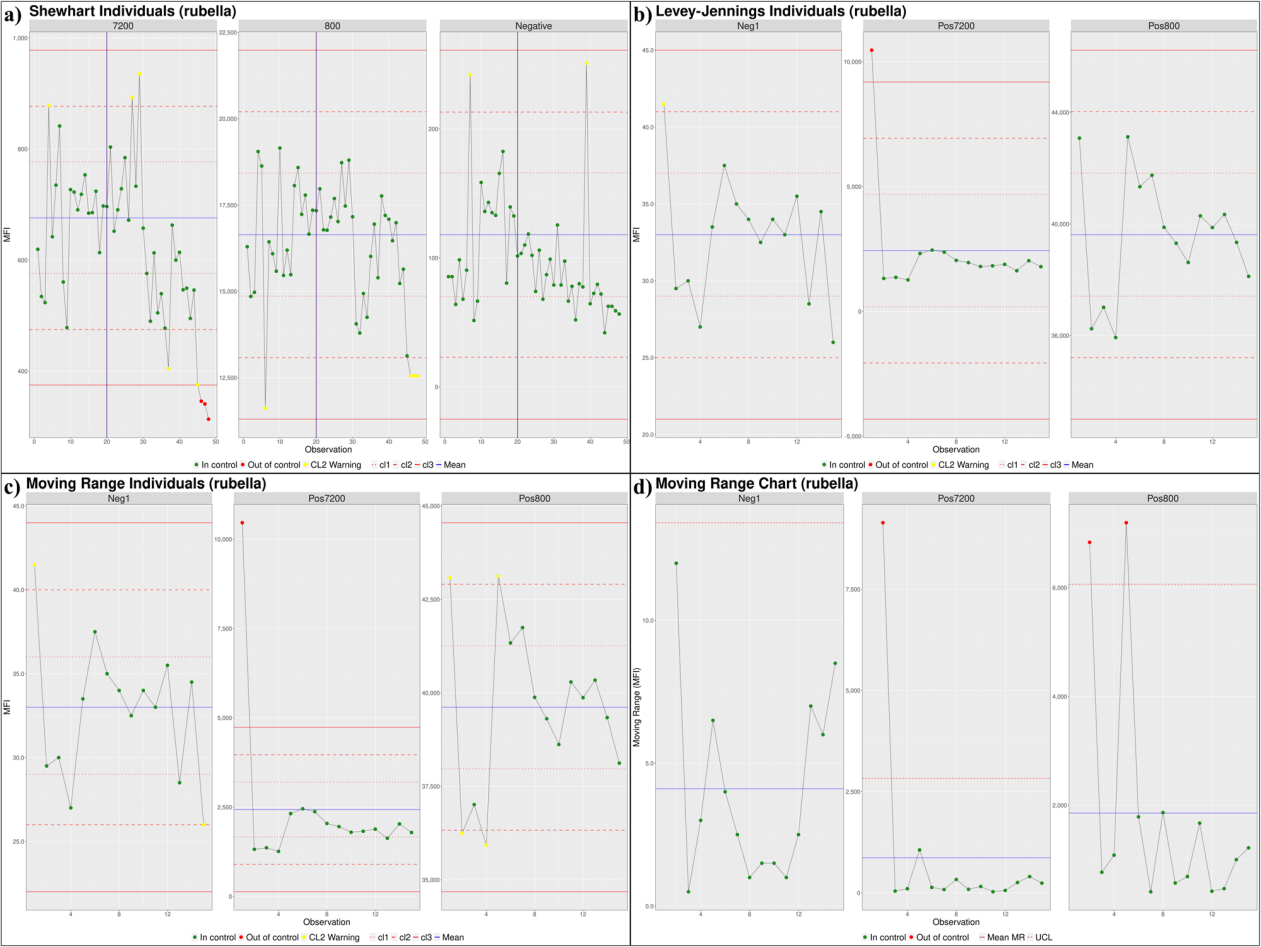
Examples of a modified Shewhart (**a**), Levey-Jennings (**b**), and Individual moving range (I-MR, **c**–**d**) control process charts of plate controls for rubella using the Guyana (**a**) and Guatemala (**b**–**d**) datasets, downloaded from shinyMBA. Shewhart control limits were defined using the initial 20 datapoints as indicated by the vertical purple line. For Shewhart, Levey-Jennings, and moving range individuals analysis (**a**–**c**), datapoints falling outside of the 3σ range were flagged as red “Out of control” and those outside of the 2σ range were flagged as yellow “CL2 Warning”. For the point-to-point moving range analysis (**d**), datapoints were flagged as red “Out of control” if |MFI moving range|≥ UCL. The figures were downloaded using the downloads module.

Following validation, the utility and output of the Control Tracking module was described using process control charts generated by shinyMBA to monitor assay controls using the datasets from Guatemala and Guyana. Control tracking output plots from shinyMBA using the rubella antigen were used as an example of the downloadable output for the control tracking module with each of the included study controls (low positive (7200), high positive (800), and negative) depicted (Fig. 7). The Shewhart chart example included Guyana plate control data for the rubella antigen visualized by run date (Fig. 7a). The Levey-Jennings chart example was generated using plate control data for the rubella antigen from the Guatemala study. It was determined that plate 1 was an outlier based on the initial process control chart in Fig. 7b. The I-MR chart example included plate control data for the rubella antigen from the Guatemala study. This method also identified plate 1 as an outlier due to elevated control MFI values (Fig. 7c–d).

The datasets from the Guyana study had a greater number of plates measured and allowed assessment of the three process control chart methods available within shinyMBA, while only the Levey-Jennings and I-MR process control methods were evaluated for the dataset from Guatemala. A total of 956 control data points in the dataset from Guyana and 300 control data points in the dataset from Guatemala were generated using three controls (neg, pos800, pos7200) included in each test plate (48 plates, Guyana study and 15 plates, Guatemala study) that evaluated 20 antigens in each dataset. Levey-Jennings charts and I-MR process control charts were generated for each dataset in shinyMBA, and the modified Shewhart chart was generated only for the dataset from Guyana only because enough plates were available for this dataset only to use the modified Shewhart method in shinyMBA. Each of the process control chart methods available in shinyMBA were compared by assessing the number of datapoints falling outside of the 3σ range (Supplementary Fig. 25). Compared to the Levey-Jennings chart and modified Shewhart chart, I-MR σ estimations resulted in tighter control limits and a higher number of flagged datapoints for each of the control (Supplementary Fig. 25). Further evaluation of the output from the shinyMBA by individual antigen and method demonstrated which antigens made up the control failures and identified antigens that demonstrated higher control failures in each study. For the dataset from Guatemala removal of plate 1, shown to be an outlier, improved the overall number of data points flagged beyond the 3σ control limit (Supplementary Figs. 26–27).

## Discussion

A platform capable of performing the complex data processing, QC analyses, and visualizations required for MBA studies is highly valuable for routine and high throughput multiplex studies. While a comprehensive Luminex QC platform does exist in the LabKey Server, its limited open-source features are not sufficient for conducting MBA QC in most laboratories^20,21^. Current open-source R Shiny web applications specific to Luminex-based assays are sparse and are not designed for conducting QC^22,23^. shinyMBA was designed to address this gap by providing an open-source suite of streamlined tools to conduct QC for studies utilizing the Luminex MBA platform, including serosurveys. This application takes advantage of the extensive computational resources of R and Shiny to provide an intuitive web-based interface that allows researchers to easily explore and analyze MBA data while minimizing the need to perform daunting, time-intensive, and error-prone data cleaning and transferring steps. In addition, shinyMBA has flexible options for customizing parameters to specific QC criteria. The capability of shinyMBA to simultaneously receive large numbers of assay output files and rapidly display dynamic outputs is made possible through shinyMBA’s reactive programming model^24^. Specifically, the use of the merged data matrix as a reactive conductor ensures that the computationally demanding process of cleaning and merging assay output files is only performed when new files are uploaded in the application. Downloading high resolution multi-faceted QC plots from shinyMBA presents another computational bottleneck that is mitigated by returning each plot in parallel rather than sequentially. The use of parallel processing drastically reduces the computation time needed to return downloadable output to user, though this may vary for users hosting the application locally on a machine with limited CPU cores.

shinyMBA has functionality for evaluating bead counts and MFI, monitoring assay control performance using statistical process control charts, and generating merged datasets from xPONENT and BPM output files. The plots generated by the bead count/MFI module allow recognition of patterns that may be viewed across the plate to identify potential reagent, loading or run errors that may occur during assay performance. For example, progressively decreasing bead counts observed in the bead fluctuation plot can indicate instrument clogging that is occurring as a plate is run.

Monitoring assay control performance is a critical QC process for MBA studies that can identify deficiencies in laboratory procedures and improve the quality of reported results. Process control charts are commonly used in laboratory settings to monitor assay control performance over time. Process control was modified by Levey and Jennings to monitor laboratory assay performance using Shewart’s process control charts with small differences in the determination of confidence limits^16,25,26^. The shinyMBA control tracking module allows user defined application of the various Levey-Jennings process control chart options. The differences in determination of confidence limits can affect the control data points flagged, with I-MR charts usually resulting in the most stringent application of the control limits. The pos7200 was flagged as a 3 σ violation in both the Levey-Jennings and I-MR user defined methods. The I-MR method flagged both remaining controls as “CL2 Warning” whereas the Levey-Jennings method flagged 1 of the 2 remaining controls as a 2σ violation. Differences in the standard deviation calculation methods requires users to carefully choose their method based on assay design.

Finally, the ability to merge multiple instrument output files into a single tabular dataset provides flexibility in conducting additional analyses as these datasets can be imported into common analytical software (e.g., R, Python, SAS, SPSS, Excel, GraphPad Prism, etc.). Algorithmically generating merged datasets bypasses labor-intensive, error-prone manual extraction of instrument output data, improving data analysis methods and reproducibility of results, especially when working with large datasets from serosurveys.

QC evaluation of large serosurveillance studies identified a gap in the ability to efficiently apply QC parameters to large datasets. shinyMBA provides a method to monitor QC in a scalable format to accommodate the multiplex benefits of Luminex technology and the high throughput capacity for serological applications. The modular organization of the application is coded to easily accommodate additional QC methods or modify existing ones. This scalability ensures that if future MBA protocols increase in complexity, any additional data analysis needs can be addressed with minor effort. New QC procedures may be implemented by developers in a straightforward fashion by conducting analyses on the application’s central merged data matrix. For interested developers, the source code and sample datasets are available at https://github.com/CDCgov/shinyMBA. There are plans to expand several aspects of shinyMBA. including, but not limited to implementing flexible options for users to define plate repeat criteria for MFI and control tracking directly in the modules; improving the user interface in the Downloads module so users have more control in how outputs are returned to them; and enabling compatibility with various database interfaces to expand data management capabilities and options for importing additional instrument outputs into the application. These additions will build upon the cohesive QC evaluation provided by shinyMBA to assess critical QC parameters within MBA workflows. In addition, the app can be easily modified to provide similar QC analysis to large datasets generated in other research areas.

### Supplementary Information

Supplementary Information.

## Data Availability

The shinyMBA source code and synthetic xPONENT files are publicly available at https://github.com/CDCgov/shinyMBA. The Guatemala and Guyana serosurveillance datasets will be published in full separately and were solely used here for demonstration purposes. shinyMBA and its source code are licensed for use under the Apache License 2.0.

## References

[1] Arnold BF (2018). Integrated serologic surveillance of population immunity and disease transmission. Emerg. Infect. Dis..

[2] Coughlin MM (2021). Development of a measles and rubella multiplex bead serological assay for assessing population immunity. J. Clin. Microbiol..

[3] Rogier EW (2018). Use of bead-based serologic assay to evaluate chikungunya virus epidemic, Haiti. Emerg. Infect. Dis..

[4] Smits GP (2012). Development of a bead-based multiplex immunoassay for simultaneous quantitative detection of igg serum antibodies against measles, mumps, rubella, and varicella-zoster virus. Clin. Vaccine Immunol..

[5] Mollema L (2014). High risk of a large measles outbreak despite 30 years of measles vaccination in The Netherlands. Epidemiol. Infect..

[6] Vos RA (2020). High varicella zoster virus susceptibility in Caribbean island populations: Implications for vaccination. Int. J. Infect. Dis..

[7] Straily A (2023). Schistosomiasis seroprevalence among children aged 0–14 years in Nigeria, 2018. Am. J. Trop. Med. Hyg..

[8] Goodhew EB (2023). Changes in trachoma indicators in Kiribati with two rounds of azithromycin mass drug administration, measured in serial population-based surveys. PLoS Negl. Trop. Dis..

[9] Cooley GM (2016). Evaluation of multiplex-based antibody testing for use in large-scale surveillance for yaws: A comparative study. J. Clin. Microbiol..

[10] Smits G (2014). Seroprevalence of rubella antibodies in The Netherlands after 32 years of high vaccination coverage. Vaccine.

[11] Vos RA (2019). Risk of Measles and diphtheria introduction and transmission on bonaire, caribbean Netherlands, 2018. Am. J. Trop. Med. Hyg.

[12] Scobie HM (2016). Tetanus immunity among women aged 15–39 years in Cambodia: A national population-based serosurvey, 2012. Clin. Vaccine Immunol..

[13] Rogier E (2019). High-throughput malaria serosurveillance using a one-step multiplex bead assay. Malar. J..

[14] van den Hoogen LL (2020). Quality control of multiplex antibody detection in samples from large-scale surveys: The example of malaria in Haiti. Sci. Rep..

[15] Chang, W., Cheng, J., Allaire, J. J., Xie, Y. & McPherson, J. *shiny: Web Application Framework for R*. 2020: https://CRAN.R-project.org/package=shiny.

[16] Levey S, Jennings ER (1950). The use of control charts in the clinical laboratory. Am. J. Clin. Pathol..

[17] Montgomery DC (2013). Introduction to Statistical Quality Control.

[18] Vagenknecht, M., Soukup, J. & Matousek, S. *Rspc: Nelson Rules for Control Charts*. 2018.

[19] Saboya-Diaz MI (2023). Lessons learned from the implementation of integrated serosurveillance of communicable diseases in the Americas. Rev. Panam Salud Publ..

[20] Eckels J (2013). Quality control, analysis and secure sharing of Luminex(R) immunoassay data using the open source LabKey Server platform. BMC Bioinform..

[21] Compare LabKey Server Editions. 2021; https://www.labkey.com/products-services/labkey-server/labkey-server-editions-feature-comparison/.

[22] Meyer TC (2020). Technical report: xMAPr—high-dynamic-range (HDR) quantification of antigen-specific antibody binding. J. Proteom..

[23] Verbist B (2019). Analyzing magnetic bead QuantiGene(R) Plex 2.0 gene expression data in high throughput mode using QGprofiler. BMC Bioinform..

[24] Reactivity: An overview. 2017 [cited 2022 2 Feb]. https://shiny.rstudio.com/articles/reactivity-overview.html. Accessed 28 June 2017.

[25] Banker CA (1980). Laboratory quality control: Use of Shewhart charts and ANOVA. Am. J. Med. Technol..

[26] Westgard JO, Westgard SA (2016). Quality control review: Implementing a scientifically based quality control system. Ann. Clin. Biochem..

