# Supplementary material for: shinyMBA: a novel R shiny application for quality control of the multiplex bead assay for serosurveillance studies
Source: Sci Rep. 2024 Mar 28;14:7442. doi: 10.1038/s41598-024-57652-4 (PMC10978933; doi:10.1038/s41598-024-57652-4)
Supplement: Supplementary file 1 — Supplementary Information. [file 41598_2024_57652_MOESM1_ESM.docx]

**Supplementary Tables**

**Supplementary Table 1.** R packages used in shinyMBA

| **Package** | **Description** | **Utilization** |
| --- | --- | --- |
| [data.table](https://cran.r-project.org/web/packages/data.table/index.html) | Expanded data.frame data structure functions for rapid aggregation and manipulation of large datasets. | Generating downloadable merged datasets and summary tables in parallel (fwrite() function) |
| [dplyr](https://cran.r-project.org/web/packages/dplyr/index.html) | Tidyverse package for manipulating and cleaning datasets | Data cleaning/computations |
| [DT](https://cran.r-project.org/web/packages/DT/index.html) | Javascript functions from the 'DataTables' library that display data objects as HTML tables. | Displaying interactive summary tables |
| [forcats](https://cran.r-project.org/web/packages/forcats/index.html) | Tidyverse package for modifying factors. | Data cleaning/computations |
| [furrr](https://cran.r-project.org/web/packages/furrr/index.html) | Implementation of 'purrr' map() functions capable of being resolved using 'future'-supported backends. | Downloading high resolution plots in parallel |
| [future](https://cran.r-project.org/web/packages/future/index.html) | A lightweight and unified Future API for sequential and parallel processing of R expression via futures | Downloading high resolution plots in parallel |
| [ggplot2](https://cran.r-project.org/web/packages/ggplot2/index.html) | Tidyverse package for creating graphics. | Displaying non-interactive plots; generating downloadable plots |
| [hablar](https://cran.r-project.org/web/packages/hablar/index.html) | Functions for converting columns to new data types and managing columns with missing values. | Data cleaning/computations |
| [lubridate](https://cran.r-project.org/web/packages/lubridate/index.html) | Tidyverse package for working with date-times and time-spans. | Data cleaning/computations |
| [naniar](https://cran.r-project.org/web/packages/naniar/index.html) | Functions for facilitating the plotting of missing values and examination of imputations. | Data cleaning/computations |
| [openxlsx](https://cran.r-project.org/web/packages/openxlsx/index.html) | Toolkit for importing and creating .xlsx files. | reading and writing .xlsx files without Java dependencies |
| [parallel](https://www.rdocumentation.org/packages/parallel/versions/3.6.2) | Toolkit for implementing parallel computation methods in R. | Downloading high resolution plots in parallel |
| [plotly](https://cran.r-project.org/web/packages/plotly/index.html) | Provides a custom interface to the 'plotly.js' JavaScript library. Allows for the creation of interactive web-friendly graphics from 'ggplot2' objects. | Displaying interactive plots |
| [purrr](https://cran.r-project.org/web/packages/purrr/index.html) | Tidyverse functional programming package for working with functions and vectors. | Data cleaning/computations |
| [Rspc](https://cran.r-project.org/web/packages/Rspc/index.html) | Toolkit for implementing statistical process control methods. | Control tracking computations |
| [scales](https://cran.r-project.org/web/packages/scales/index.html) | Toolkit for customizing transformations, breaks, guides, and palettes in ggplot2 graphics. | Modifying display and downloadable plots |
| [shiny](https://cran.r-project.org/web/packages/shiny/index.html) | Framework for developing web applications in R | Development of the user interface and backend server framework |
| [shinyalert](https://cran.r-project.org/web/packages/shinyalert/index.html) | Tools for creating popup messages (modals) in Shiny. | User interface development |
| [shinycssloaders](https://cran.r-project.org/web/packages/shinycssloaders/index.html) | Tools for implementing CSS loading animations in Shiny. | User interface development |
| [shinyjs](https://cran.r-project.org/web/packages/shinyjs/index.html) | Allows for common Javascript operations in Shiny without the need for Javascript coding. | User interface development |
| [shinythemes](https://cran.r-project.org/web/packages/shinythemes/index.html) | Pre-made CSS themes for Shiny applications. | User interface development |
| [shinyWidgets](https://cran.r-project.org/web/packages/shinyWidgets/index.html) | Expanded Shiny user interface widgets and components | User interface development |
| [stringr](https://cran.r-project.org/web/packages/stringr/index.html) | Tidyvers package for string manipulation. | Data cleaning/computations |
| [tidyr](https://cran.r-project.org/web/packages/tidyr/index.html) | Tidyverse package for wrangling data into a 'tidy' format. | Data cleaning/computations |
| [zip](https://cran.r-project.org/web/packages/zip/index.html) | Cross-platform file compression functions. | Returning downloadable outputs to user |

**Supplementary Table 2.** Antigens used in the Guatemala and Guyana surveillance studies

| **Group** | **Antigen** | **Description (organism)** |
| --- | --- | --- |
| Neglected Tropical Diseases (NTDs) | pgp3 | Trachoma (*Chlamydia Trachomatis*) |
|  | CT694 |  |
|  | Bm14 | Lymphatic filariasis (*Wuchereria bancrofti*) |
|  | Bm33 |  |
|  | Wb123 |  |
|  | Ov16 | Onchocerciasis (*Onchocerca volvulus*) |
|  | OV33 |  |
| Vaccine Preventable Diseases (VPDs) | whole Measles virus | Measles (*Morbillivirus measles virus*) |
|  | whole Rubella virus | Rubella (*Rubivirus rubella virus*) |
|  | Tetanus Toxoid | Tetanus (*Clostridium tetani*) |
|  | Diphtheria Toxoid | Diphtheria (*Corynebacterium diphtheriae*) |
| Malaria | pfMSP1-19 | Malaria (*Plasmodium falciparum*) |
|  | pvMSP1-19 | Malaria (*Plasmodium vivax*) |
| Soil-transmitted Helminths (STH) | T24H | Cysticercosis (*Taenia solium*) |
|  | ES33 | Taeniasis (*Taenia solium*) |
|  | NIE | Strongyloidiasis (*Strongyloides steracoralis*) |
| Water, sanitation, and hygiene (WASH) | VSP3 | Giardiasis (*Giardia lamblia*) |
|  | VSP5 |  |
| Negative Controls | GST | Glutathione-s-transferase (N/A) |
|  | Vero | Vero-cell lysate (N/A) |

**Supplementary Figures**


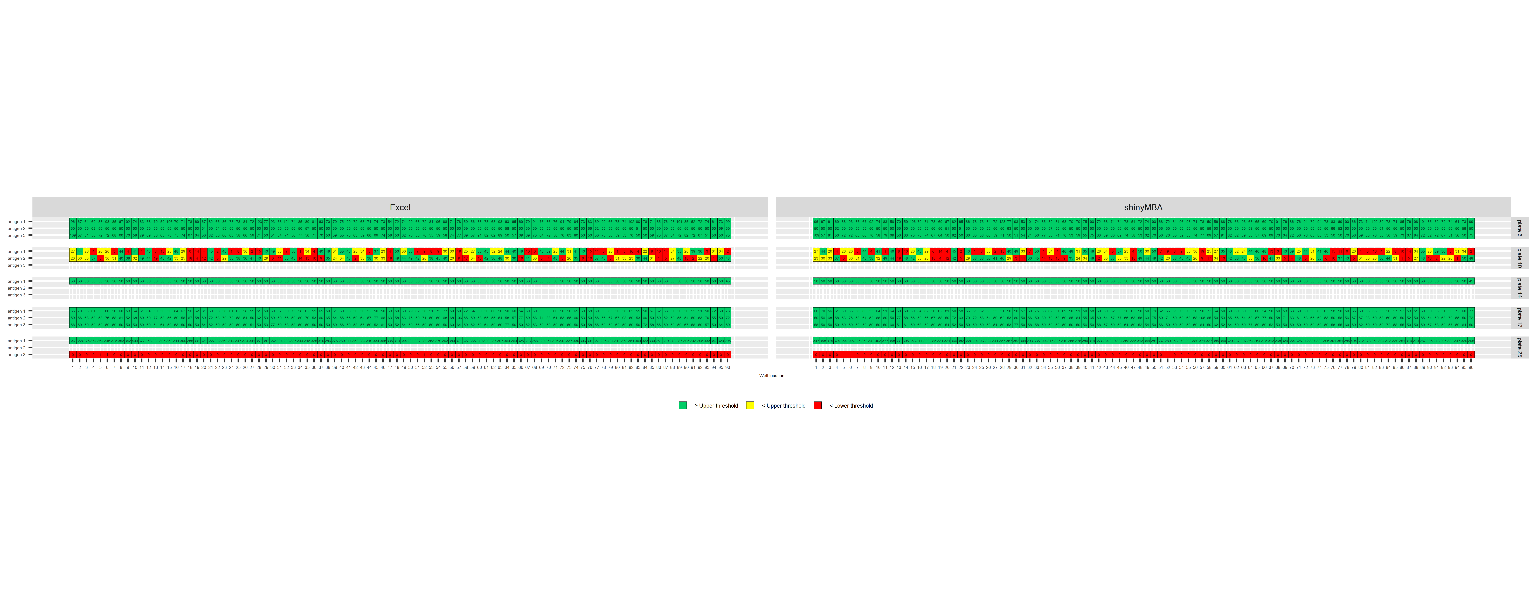


**Supplemental Figure 1.** Bead count flagging validation results using the default shinyMBA bead count thresholds. Each tile represents a single well-antigen combination with the bead count included as text. The x-axis represents the chronological order that the wells were read. Bead counts below the lower threshold (20 beads/well) were flagged as red while those between the lower and upper (35 beads/well) thresholds were flagged as yellow. Any bead count at or above the upper threshold was flagged as green.


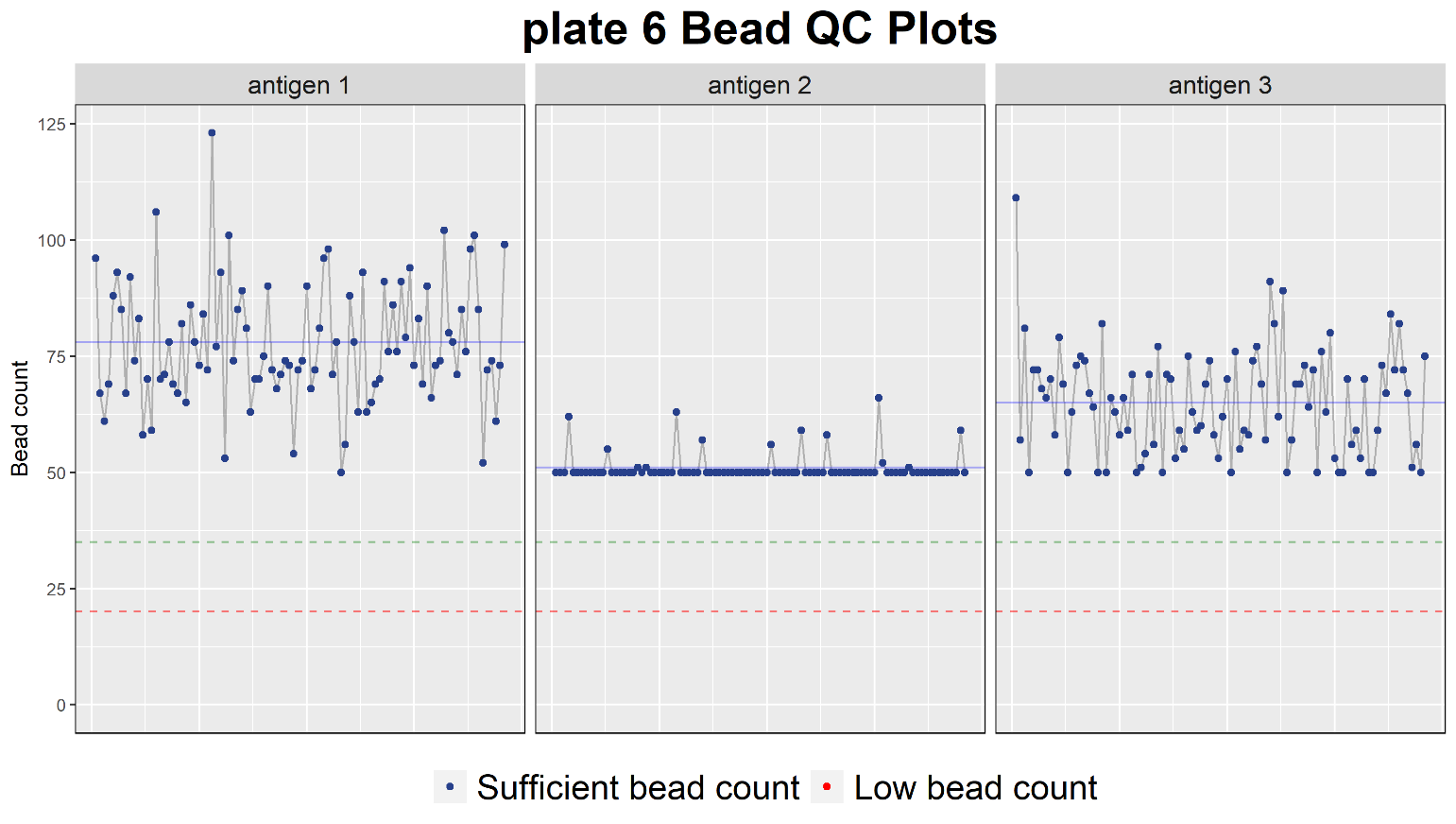


**Supplementary Figure 2.** shinyMBA bead count fluctuation plots for plate 6. The bead count upper threshold was set at 35 beads/well (green reference line) and the lower threshold was set at 20 beads/well (red reference line). The blue reference line indicates the mean bead count. The x axis represents individual plate wells by instrument read order. Samples with bead counts under the lower threshold were visualized as red points on the plot.


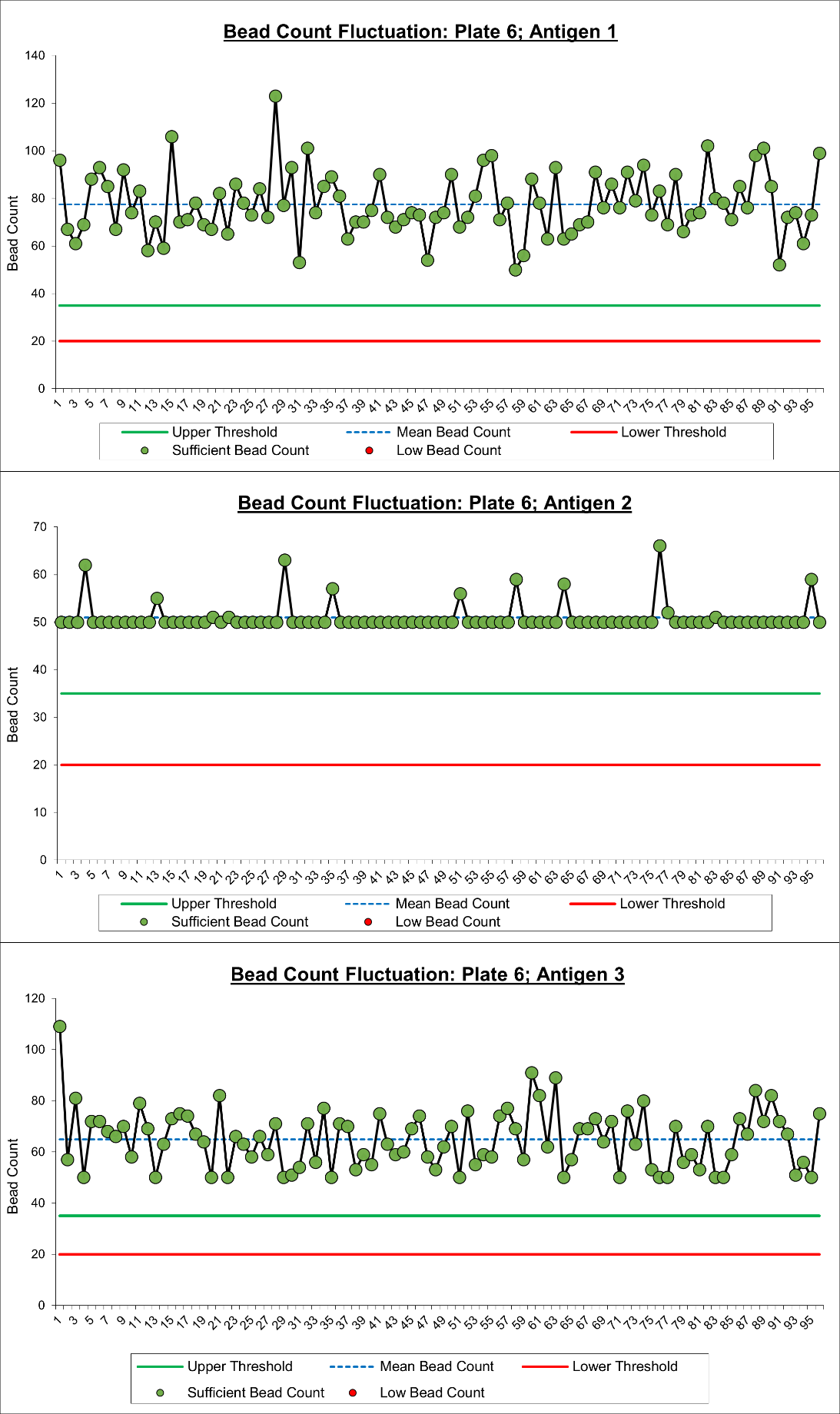


**Supplementary Figure 3.** Excel bead count fluctuation plots for plate 6. The bead count upper threshold was set at 35 beads/well (green reference line) and the lower threshold was set at 20 beads/well (red reference line). The x axis represents individual plate wells by instrument read order. Samples with bead counts under the lower threshold were visualized as red points on the plot.


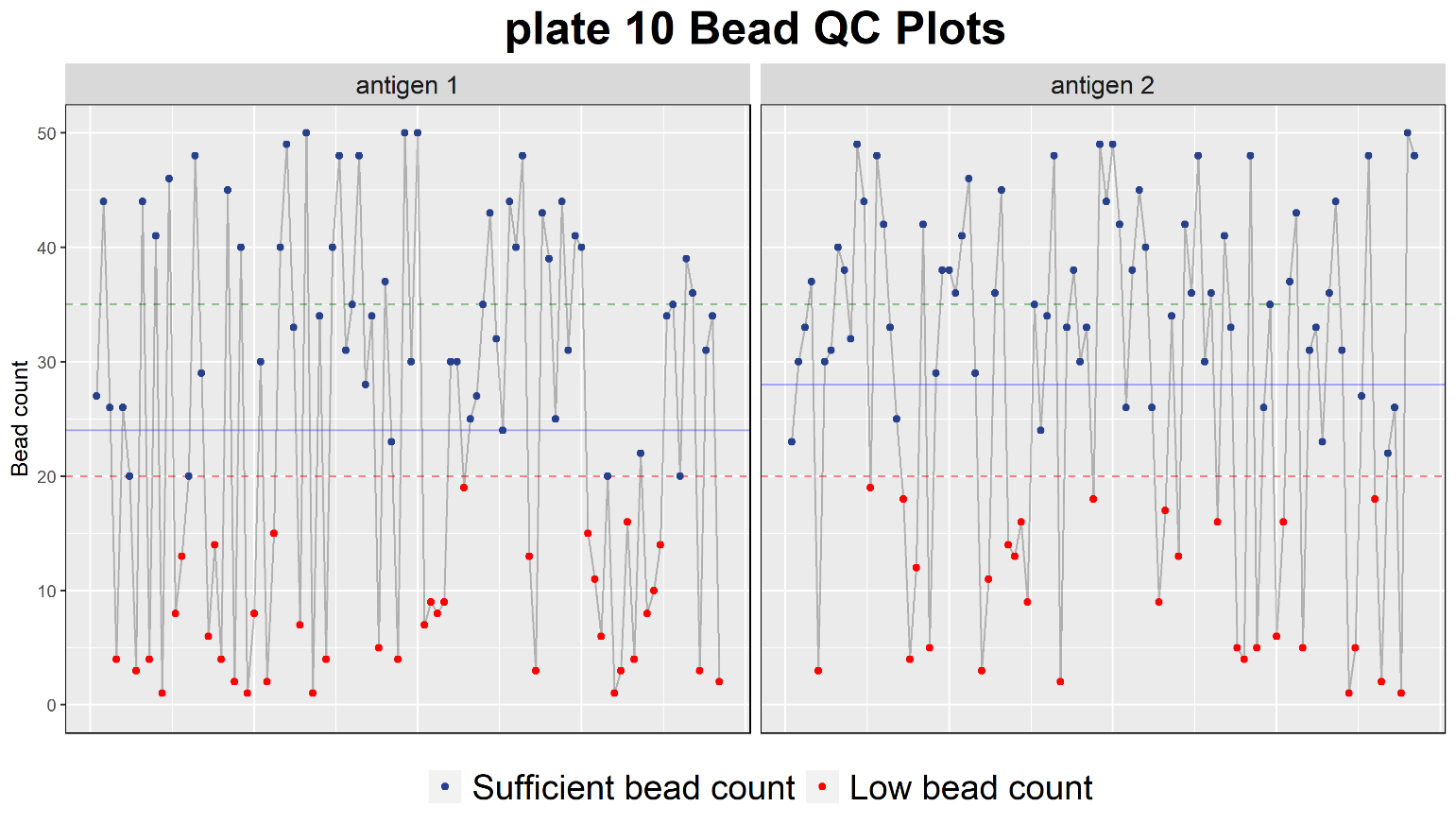


**Supplementary Figure 4.** shinyMBA bead count fluctuation plots for plate 10. The bead count upper threshold was set at 35 beads/well (green reference line) and the lower threshold was set at 20 beads/well (red reference line). The blue reference line indicates the mean bead count. The x axis represents individual plate wells by instrument read order. Samples with bead counts under the lower threshold were visualized as red points on the plot.


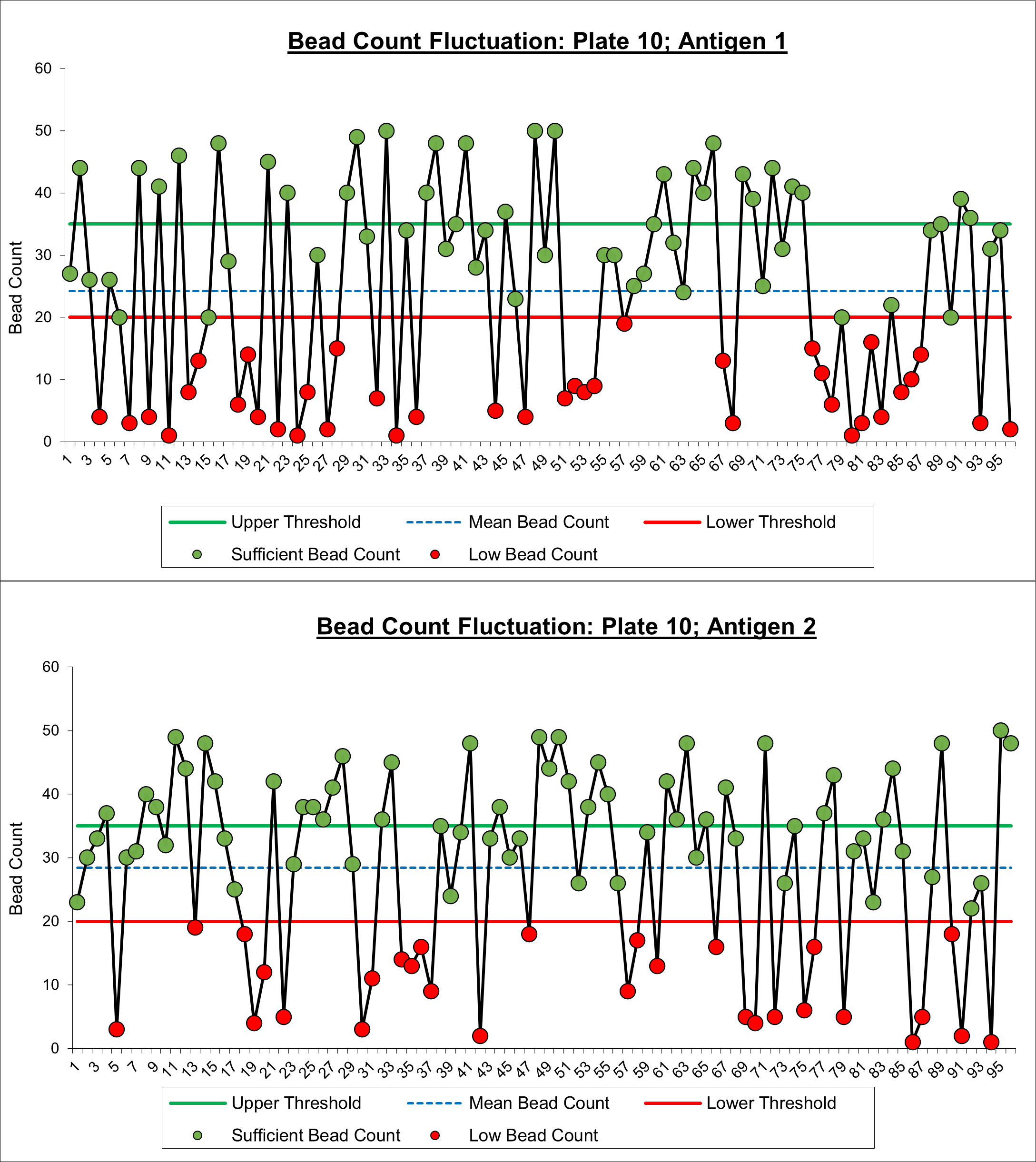


**Supplementary Figure 5.** Excel bead count fluctuation plots for plate 10. The bead count upper threshold was set at 35 beads/well (green reference line) and the lower threshold was set at 20 beads/well (red reference line). Samples with bead counts under the lower threshold were visualized as red points on the plot.


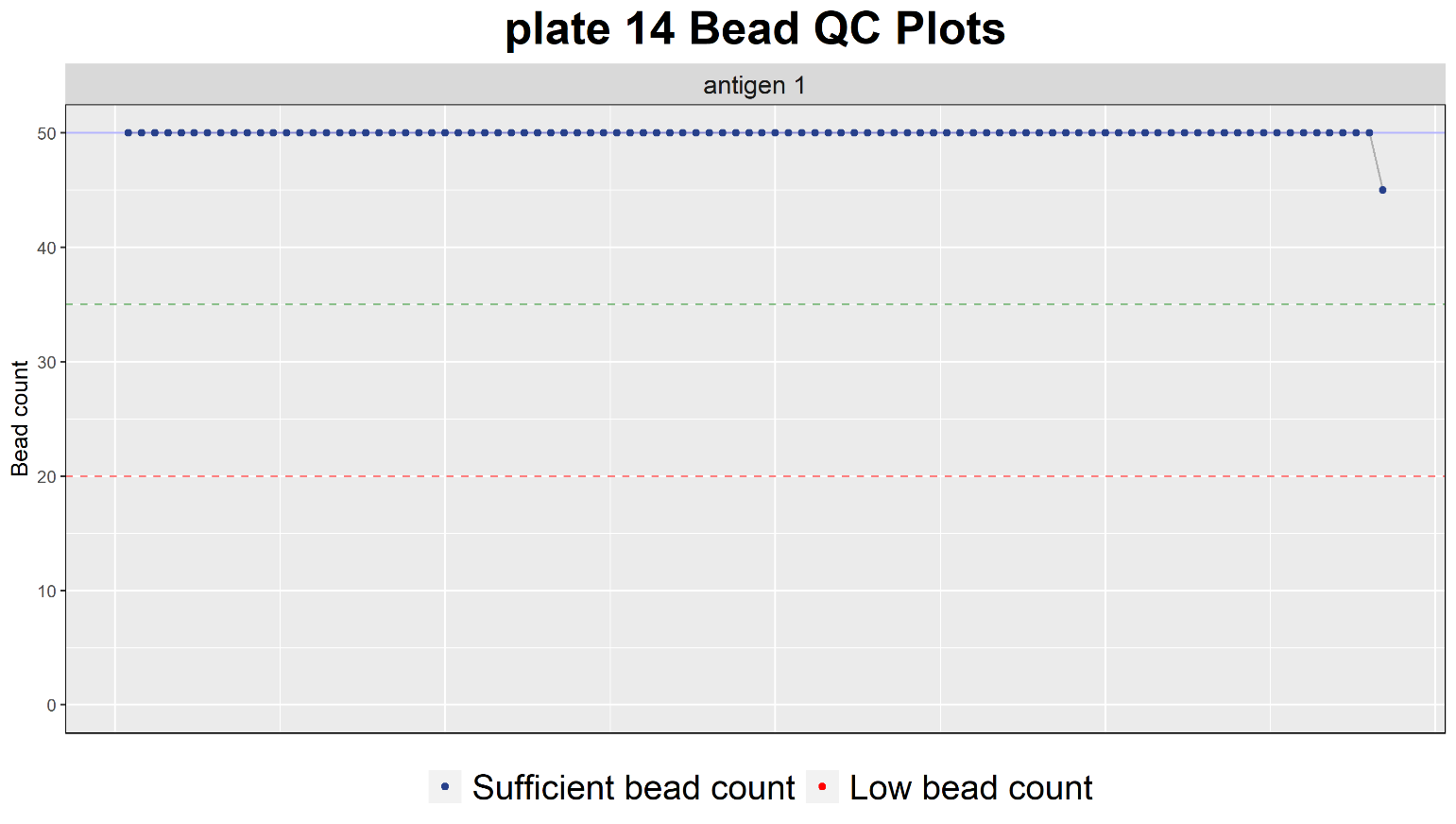


**Supplementary Figure 6.** shinyMBA bead count fluctuation plot for plate 14. The bead count upper threshold was set at 35 beads/well (green reference line) and the lower threshold was set at 20 beads/well (red reference line). The blue reference line indicates the mean bead count. The x axis represents individual plate wells by instrument read order. Samples with bead counts under the lower threshold were visualized as red points on the plot.


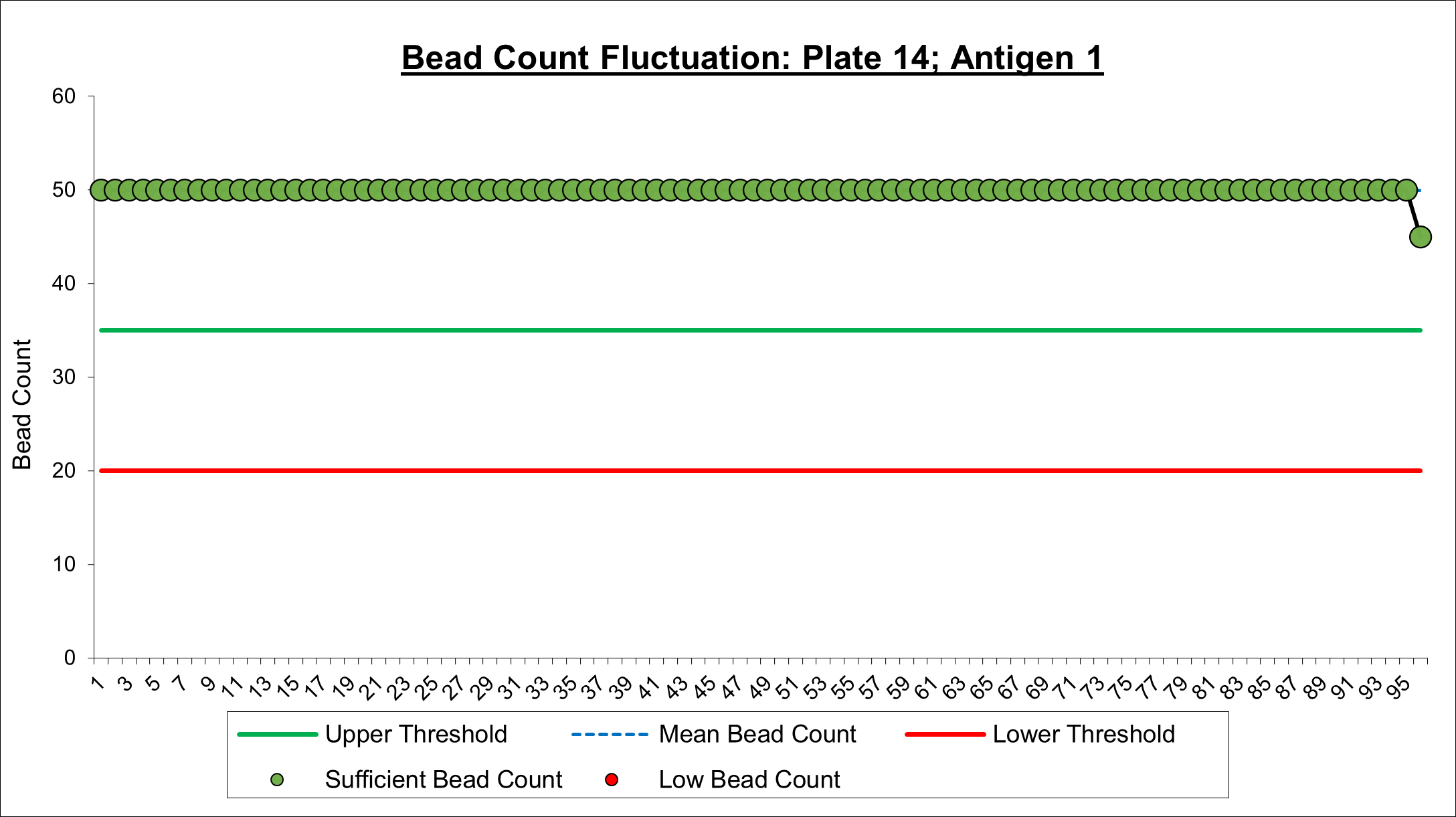


**Supplementary Figure 7.** Excel bead count fluctuation plots for plate 14. The bead count upper threshold was set at 35 beads/well (green reference line) and the lower threshold was set at 20 beads/well (red reference line). The x axis represents individual plate wells by instrument read order. Samples with bead counts under the lower threshold were visualized as red points on the plot.


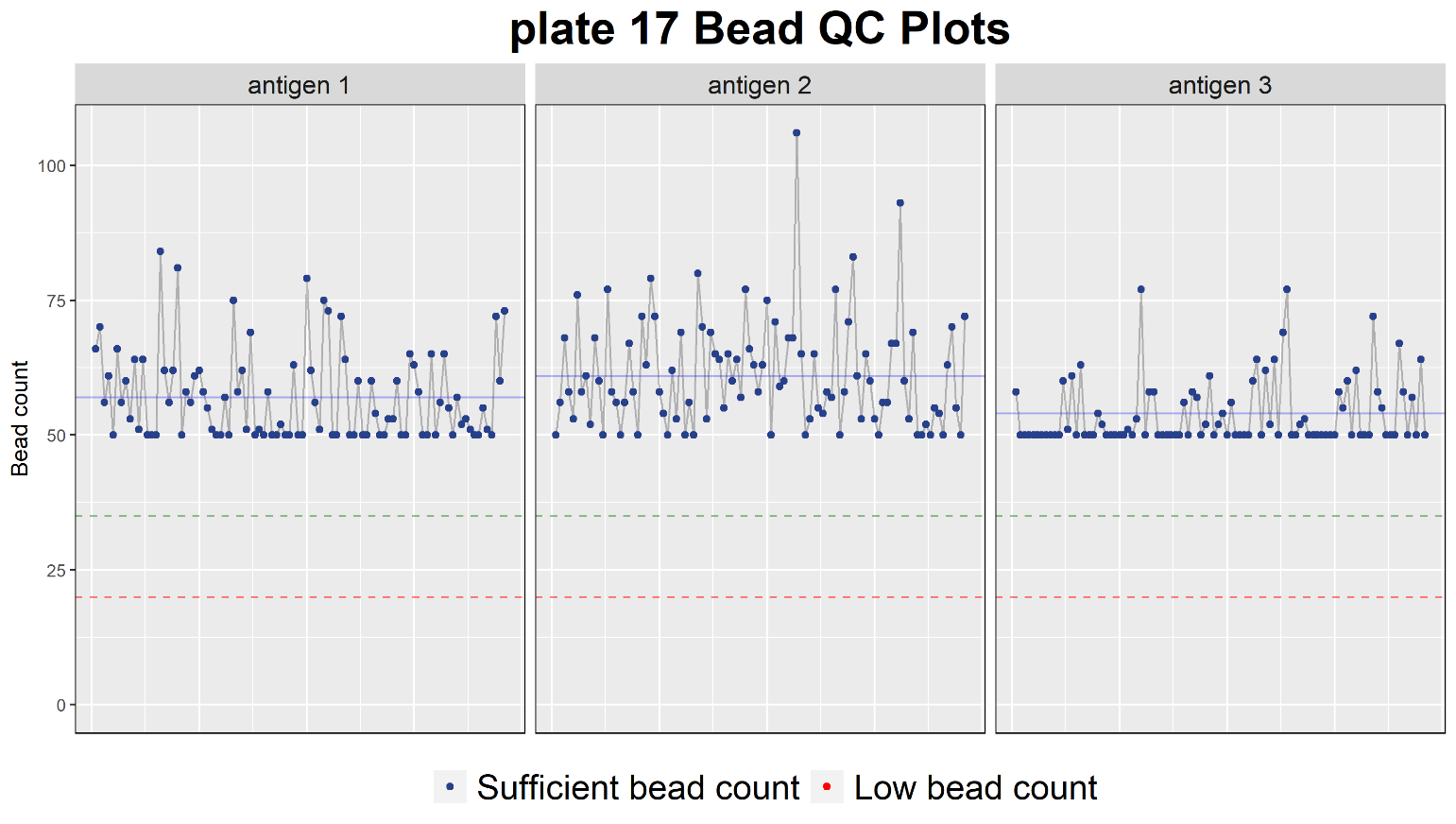


**Supplementary Figure 8.** shinyMBA bead count fluctuation plots for plate 17. The bead count upper threshold was set at 35 beads/well (green reference line) and the lower threshold was set at 20 beads/well (red reference line). The blue reference line indicates the mean bead count. The x axis represents individual plate wells by instrument read order. Samples with bead counts under the lower threshold were visualized as red points on the plot.


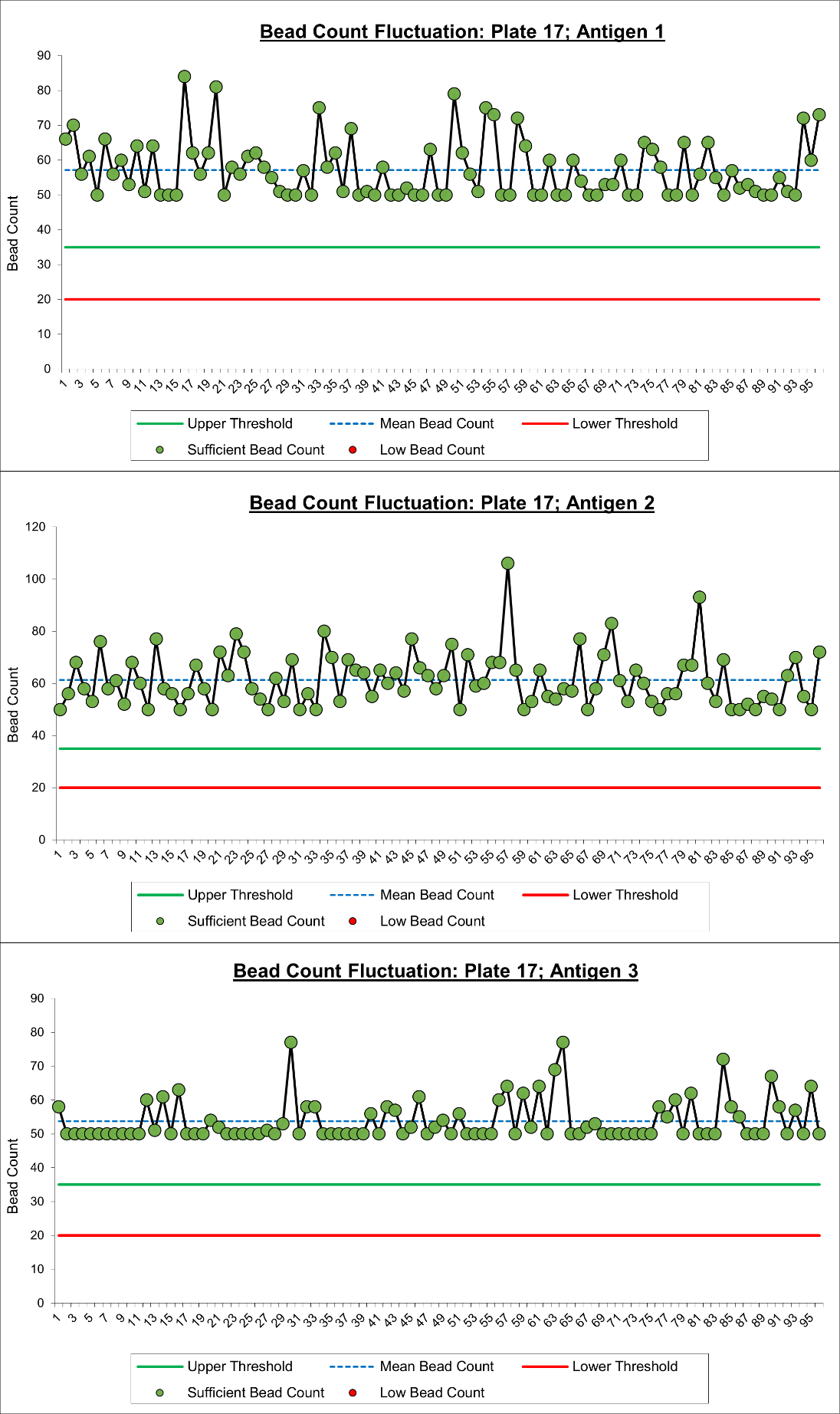


**Supplementary Figure 9.** Excel bead count fluctuation plots for plate 17. The bead count upper threshold was set at 35 beads/well (green reference line) and the lower threshold was set at 20 beads/well (red reference line). The x axis represents individual plate wells by instrument read order. Samples with bead counts under the lower threshold were visualized as red points on the plot.


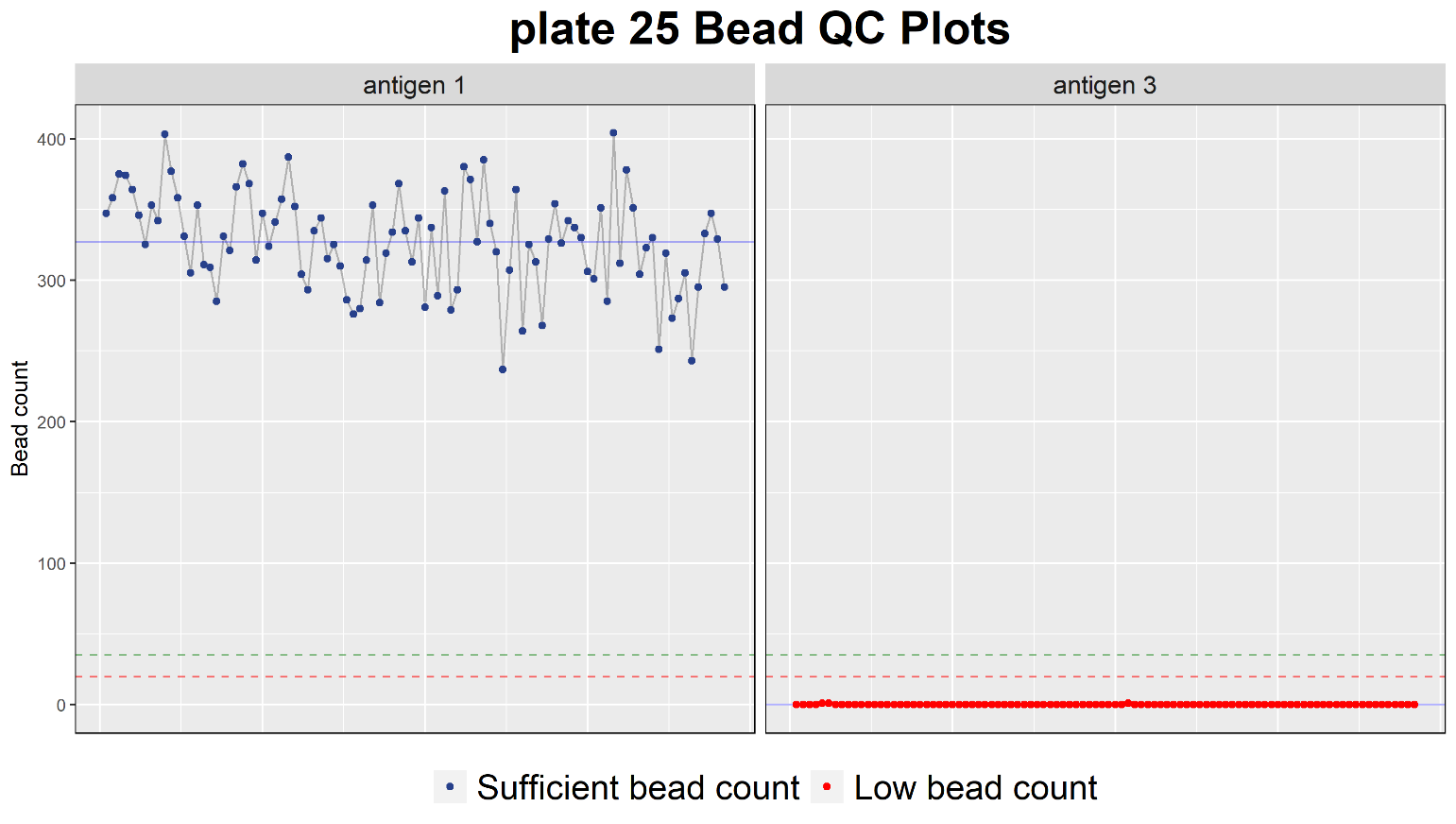


**Supplementary Figure 10.** shinyMBA bead count fluctuation plots for plate 25. The bead count upper threshold was set at 35 beads/well (green reference line) and the lower threshold was set at 20 beads/well (red reference line). The blue reference line indicates the mean bead count. The x axis represents individual plate wells by instrument read order. Samples with bead counts under the lower threshold were visualized as red points on the plot.


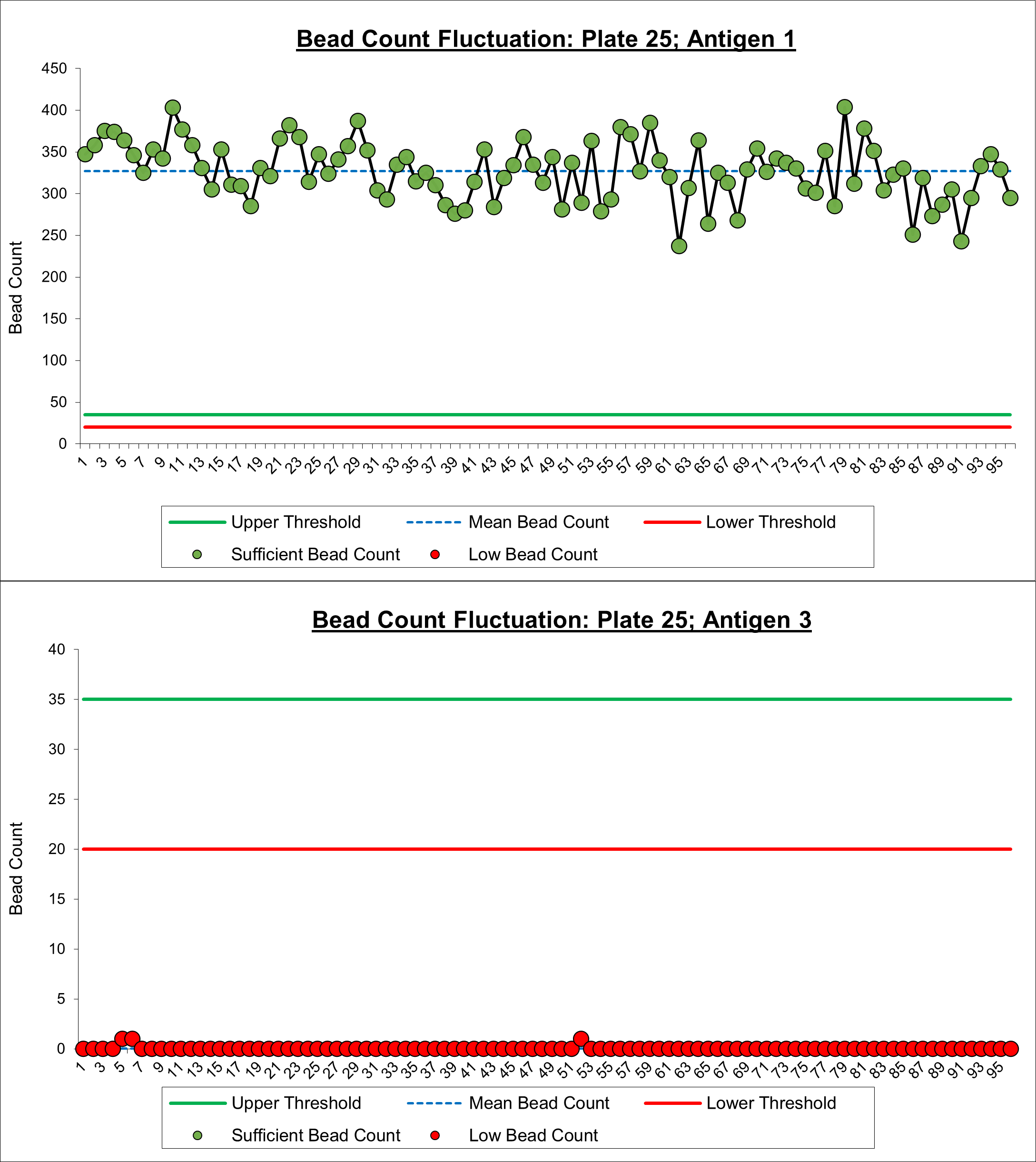


**Supplementary Figure 11.** Excel bead count fluctuation plots for plate 25. The bead count upper threshold was set at 35 beads/well (green reference line) and the lower threshold was set at 20 beads/well (red reference line). The x axis represents individual plate wells by instrument read order. Samples with bead counts under the lower threshold were visualized as red points on the plot.


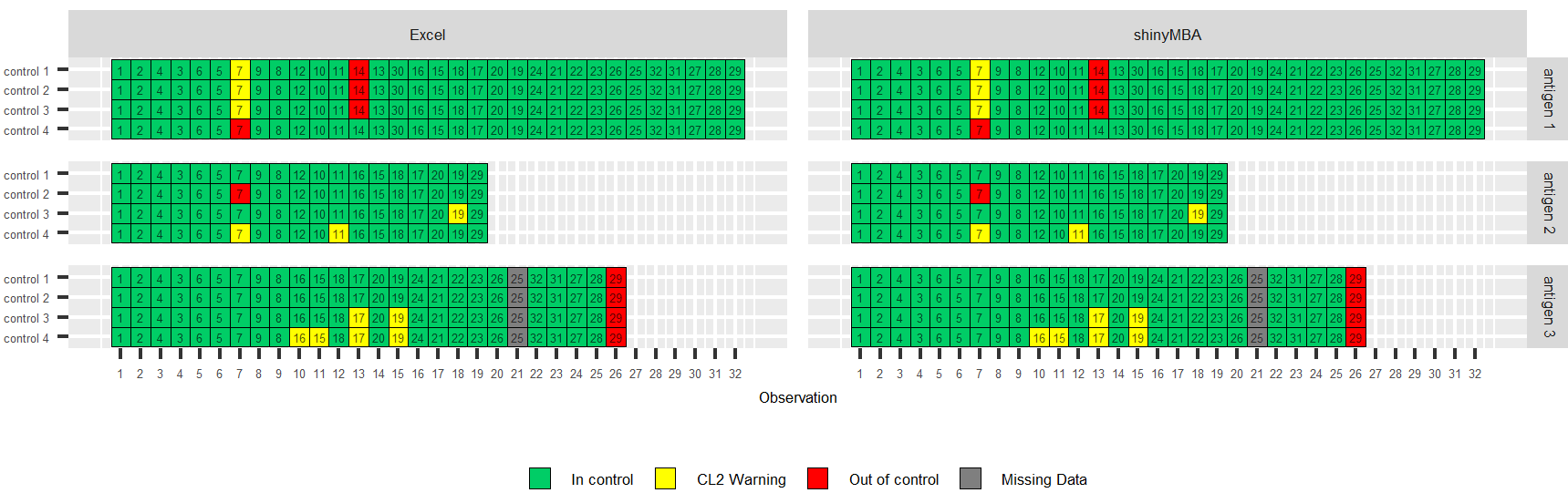


**Supplemental Figure 12.** I-MR control tracking validation results using 2CL and 3CL datapoint flagging faceted by method used and antigen name. Each tile represents a single plate-control combination with the plate number included as text. The x-axis represents the chronological order that the plates were read. Datapoints falling outside of the 3σ range were flagged as red “Out of control” and those outside of the 2σ range were flagged as yellow “CL2 Warning”.


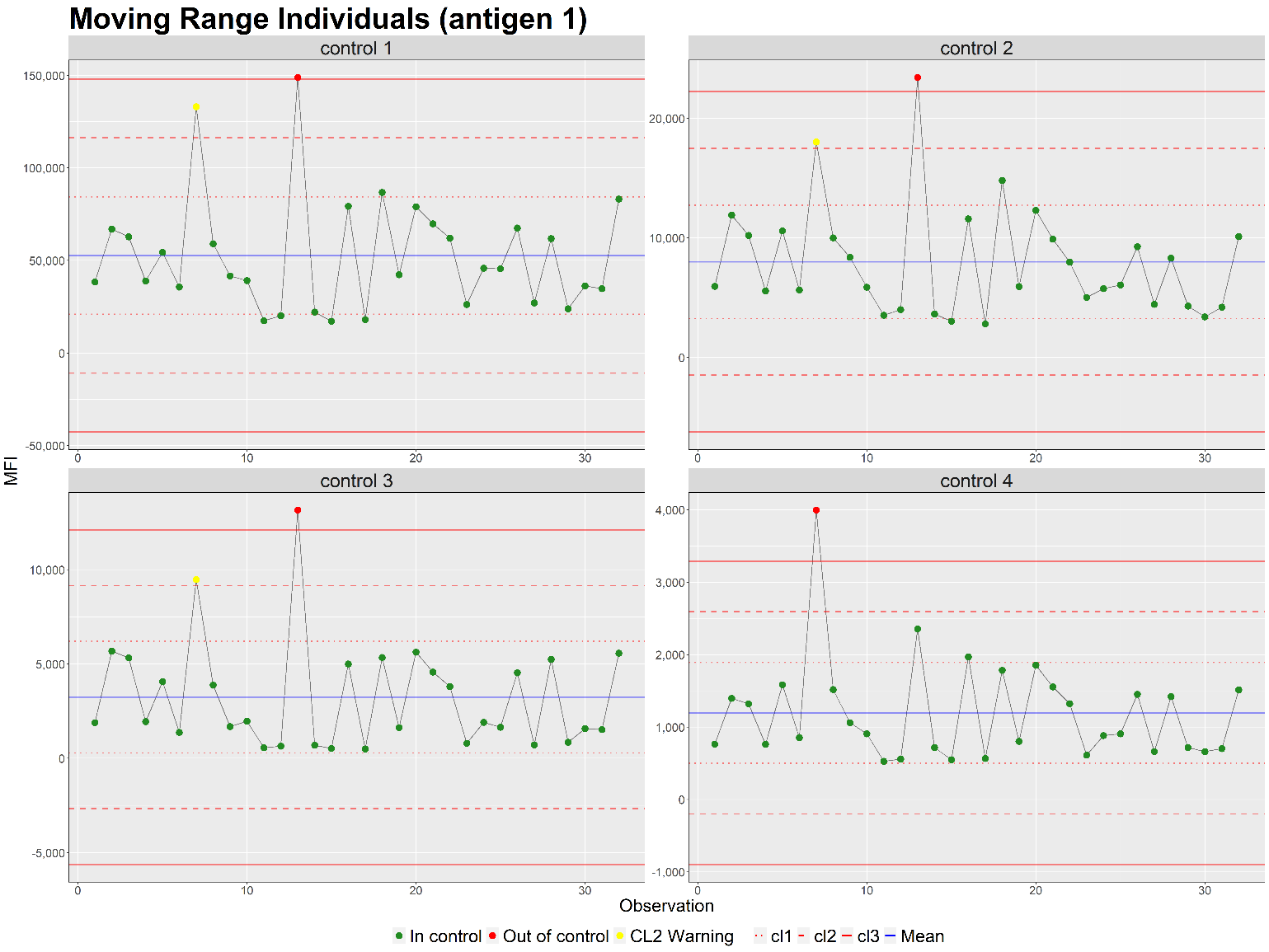


**Supplementary Figure 13.** shinyMBA MFI moving range individuals chart for antigen 1. Datapoints falling outside of the 3σ range were flagged as red “Out of control” and those outside of the 2σ range were flagged as yellow “CL2 Warning”.


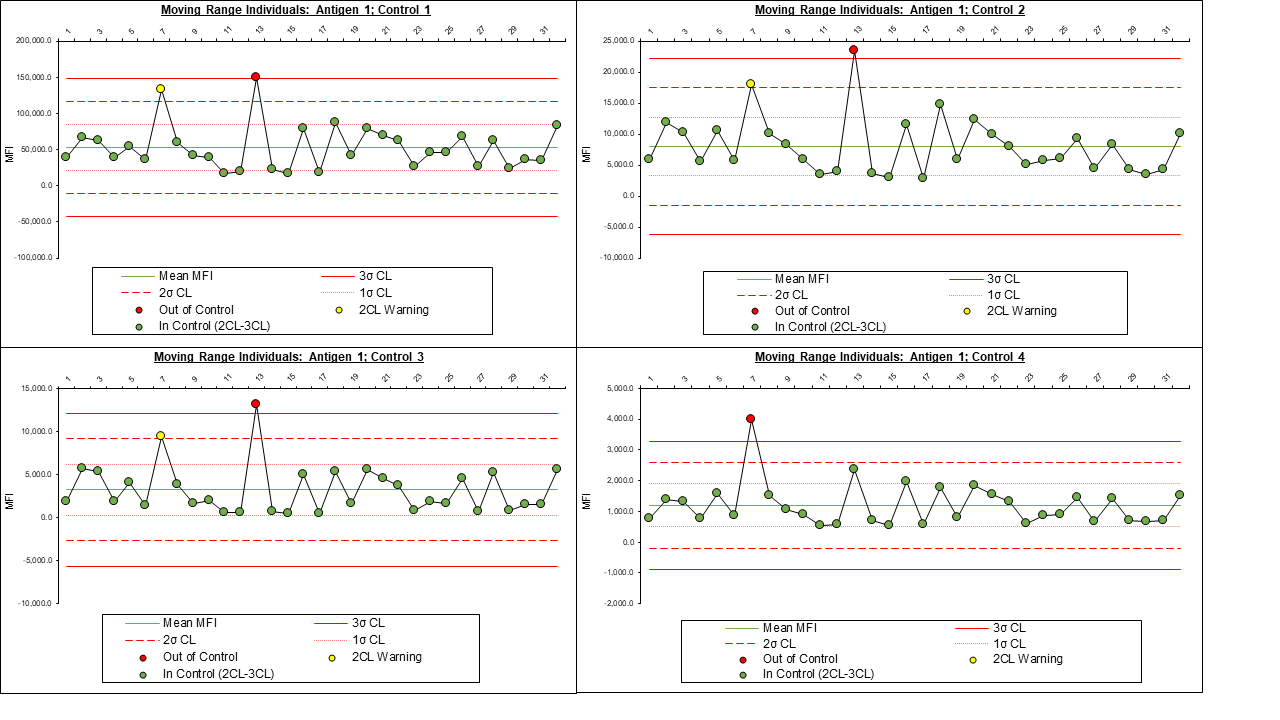


**Supplementary Figure 14.**  Excel MFI moving range individuals charts for antigen 1. Datapoints falling outside of the 3σ range were flagged as red “Out of control” and those outside of the 2σ range were flagged as yellow “CL2 Warning”.


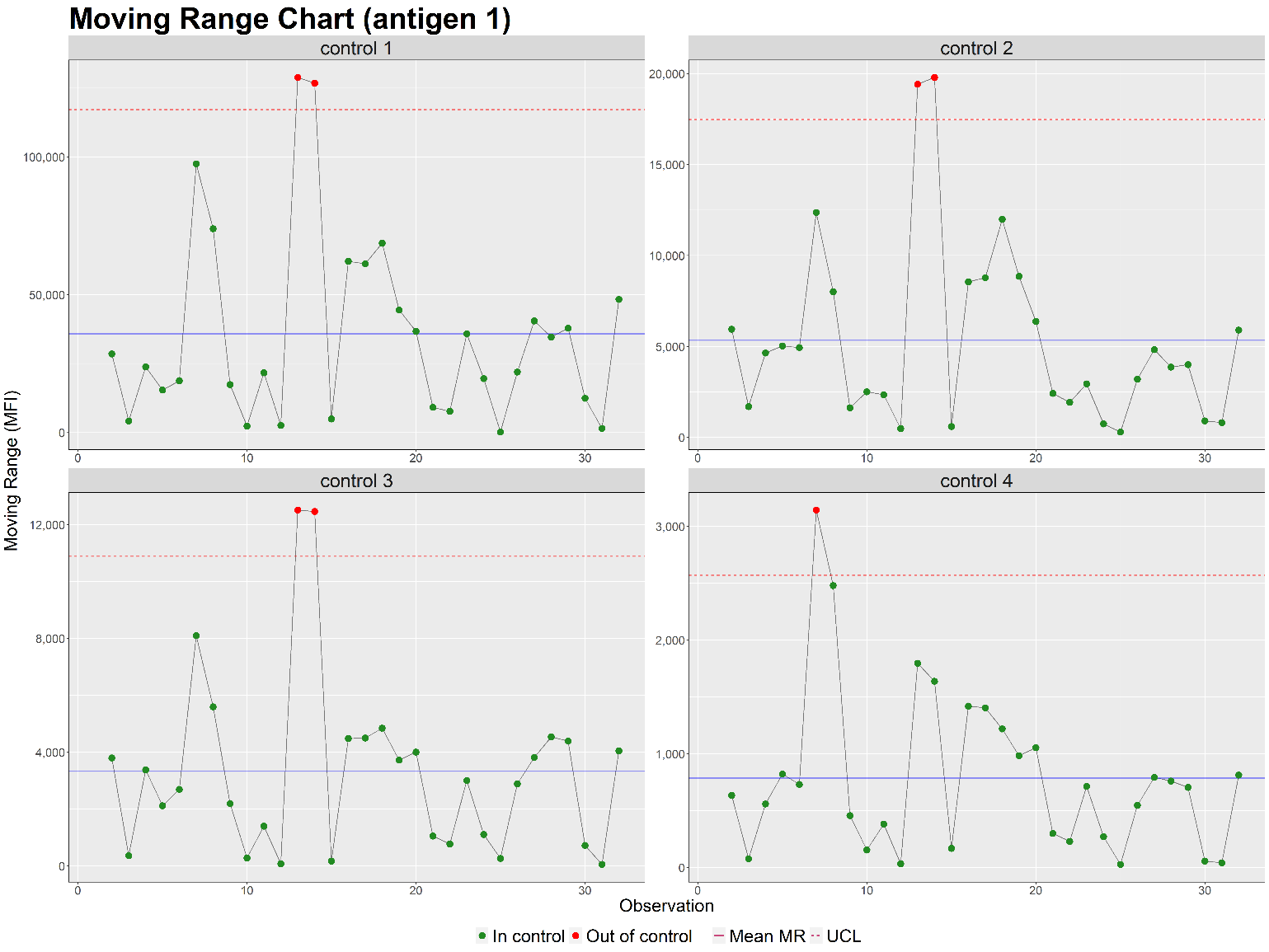


**Supplementary Figure 15.** shinyMBA MFI moving range charts for antigen 1. Datapoints falling outside of the moving range upper confidence limit (UCL) were flagged as red “Out of Control”.


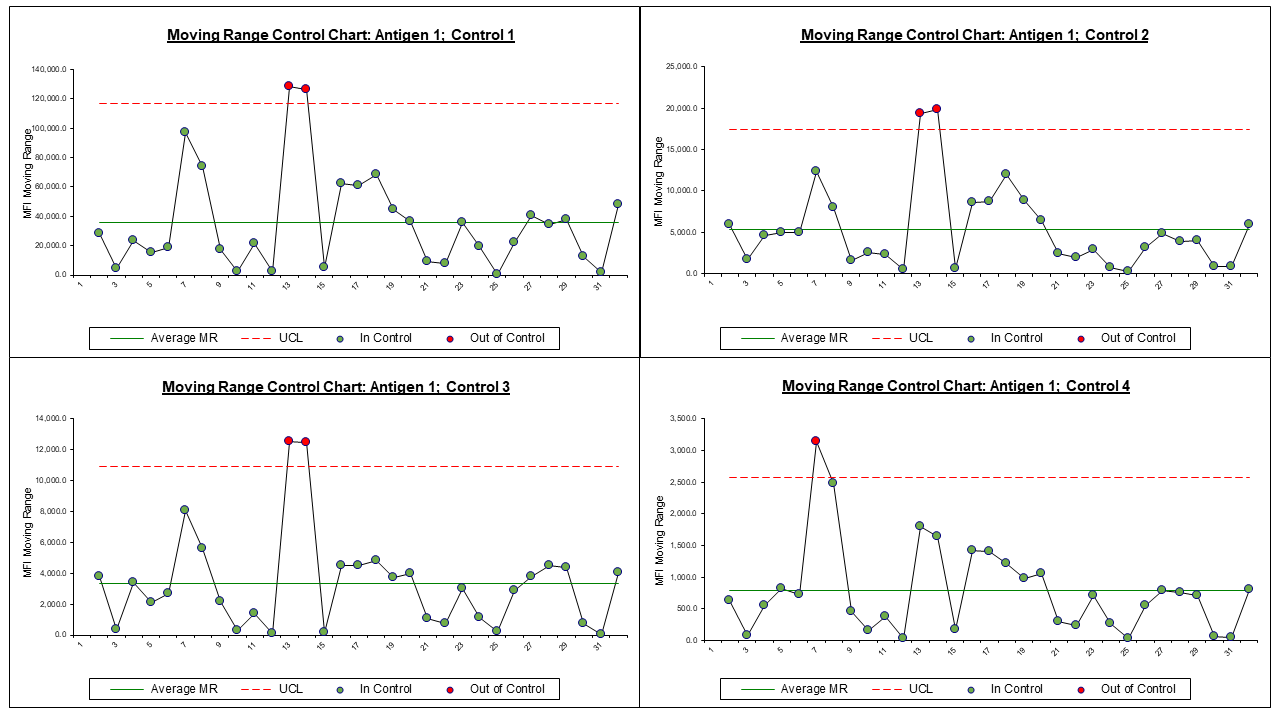


**Supplementary Figure 16**. Excel MFI moving range charts for antigen 1. Datapoints falling outside of the moving range upper confidence limit (UCL) were flagged as red “Out of Control”.


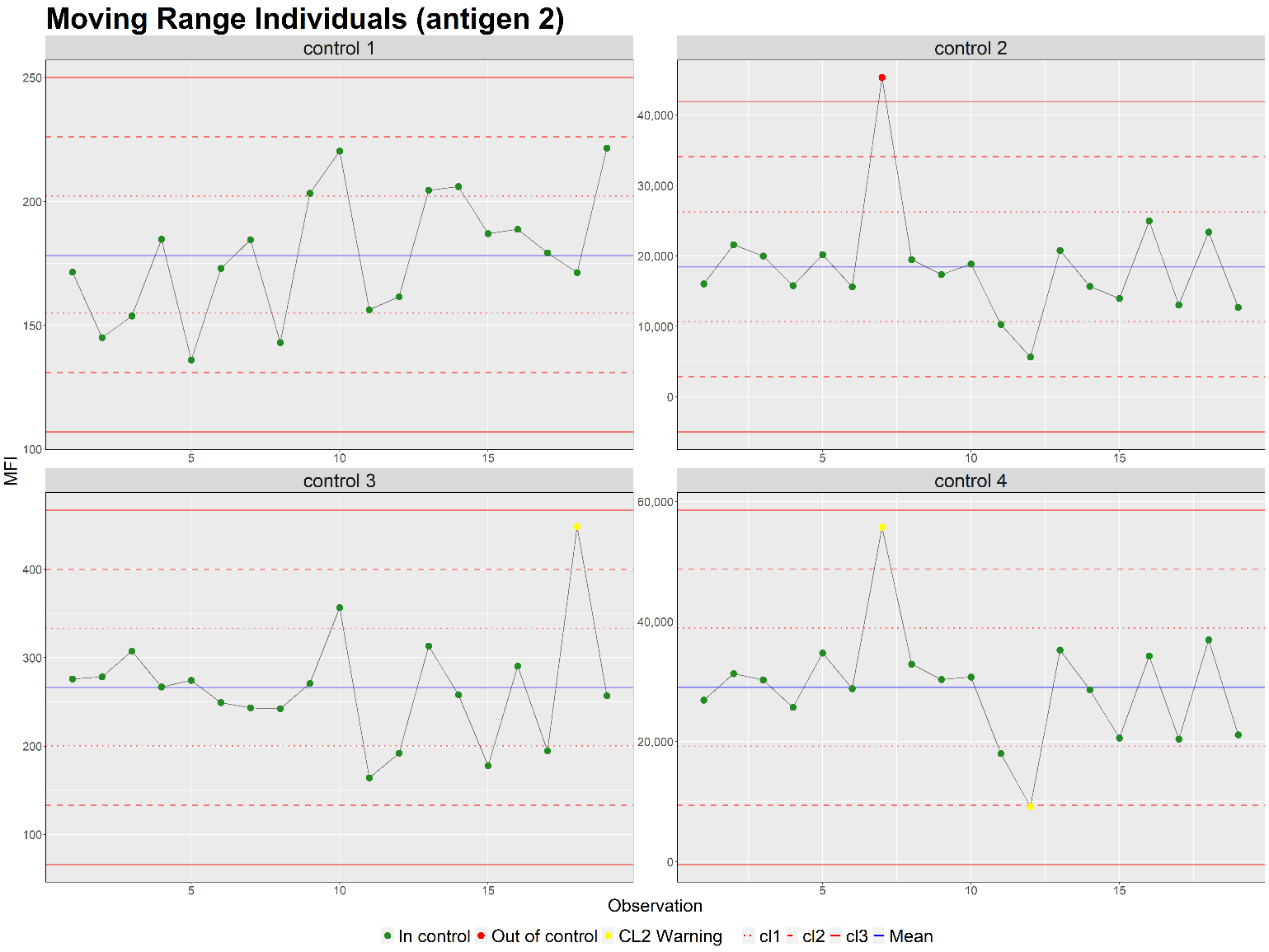


**Supplementary Figure 17.** shinyMBA MFI moving range individuals charts for antigen 2. Datapoints falling outside of the 3σ range were flagged as red “Out of control” and those outside of the 2σ range were flagged as yellow “CL2 Warning”.


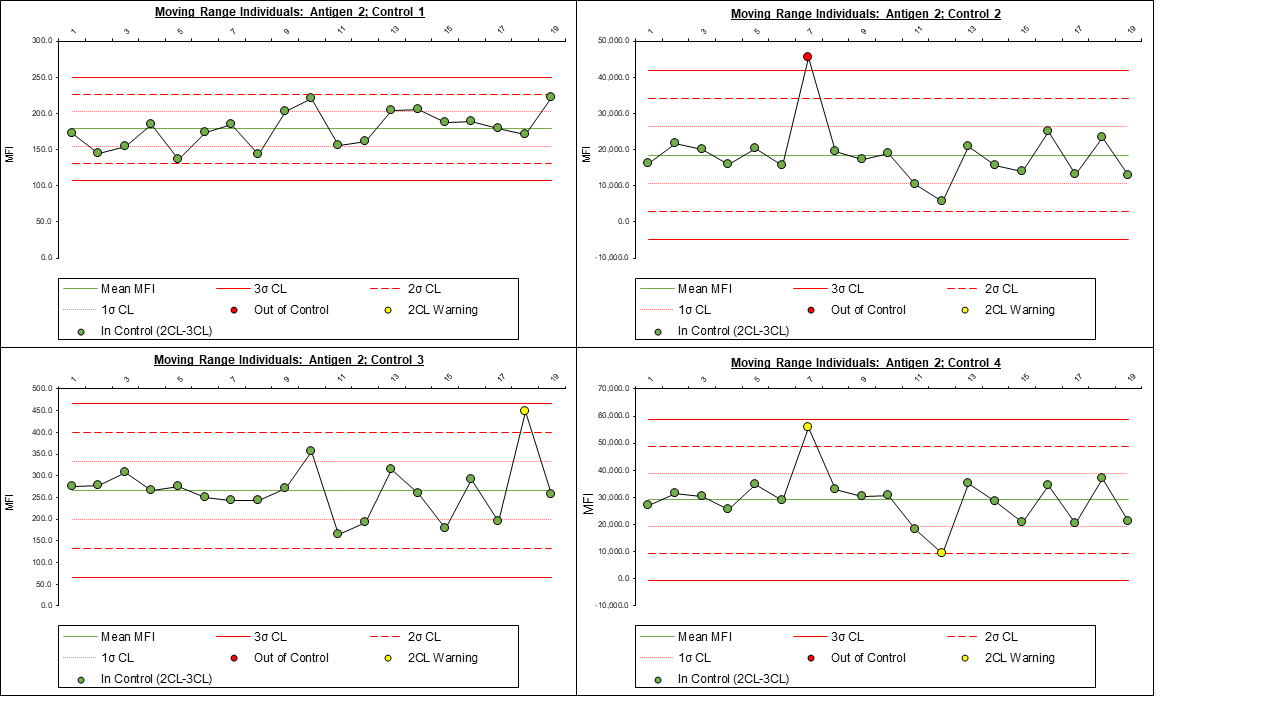


**Supplementary Figure 18.** Excel MFI moving range individuals charts for antigen 2. Datapoints falling outside of the 3σ range were flagged as red “Out of control” and those outside of the 2σ range were flagged as yellow “CL2 Warning”.


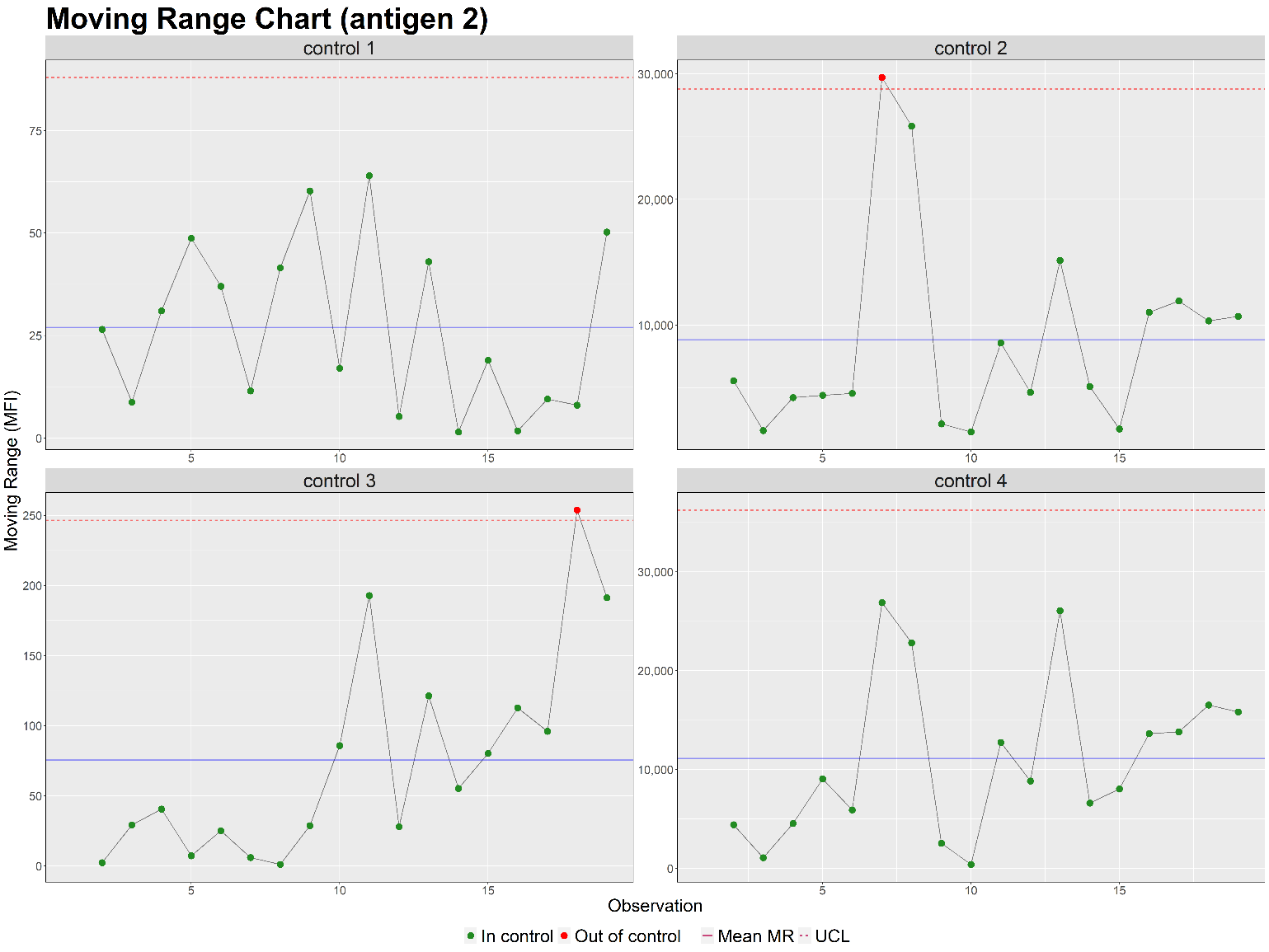


**Supplementary Figure 19.** shinyMBA MFI moving range charts for antigen 2. Datapoints falling outside of the moving range upper confidence limit (UCL) were flagged as red “Out of Control”.


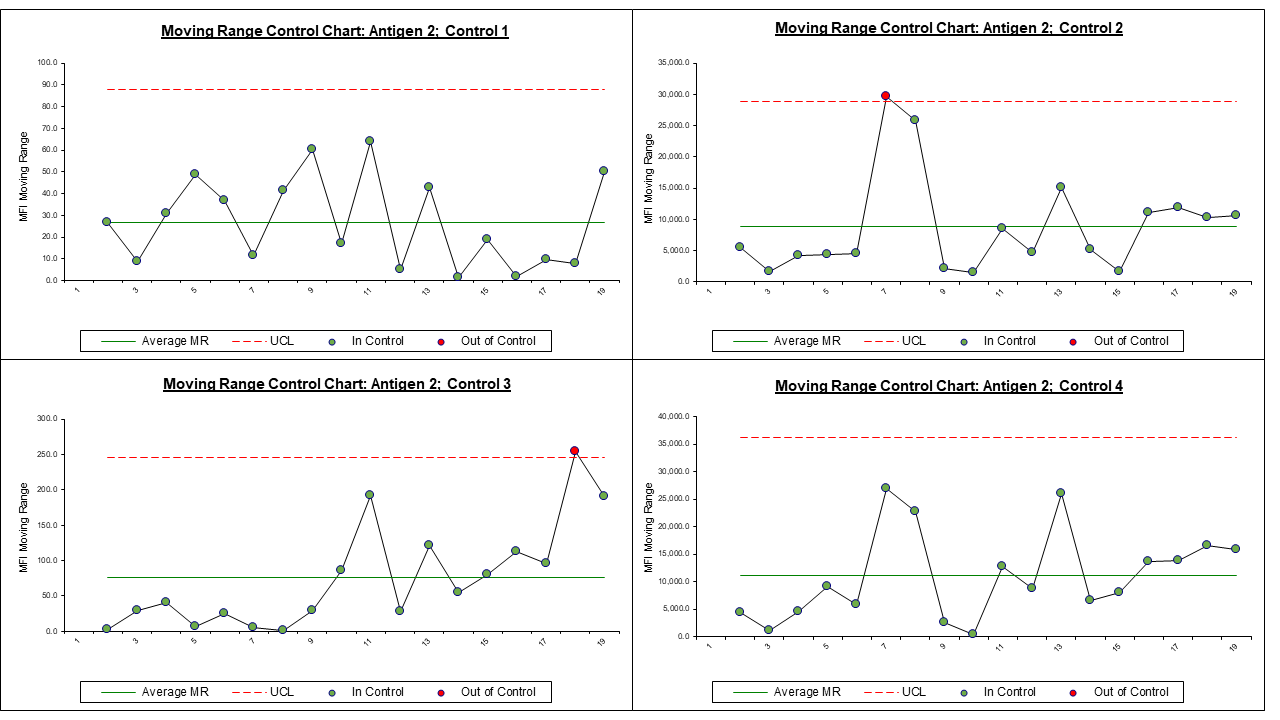


**Supplementary Figure 20.** Excel MFI moving range charts for antigen 2. Datapoints falling outside of the moving range upper confidence limit (UCL) were flagged as red “Out of Control”.


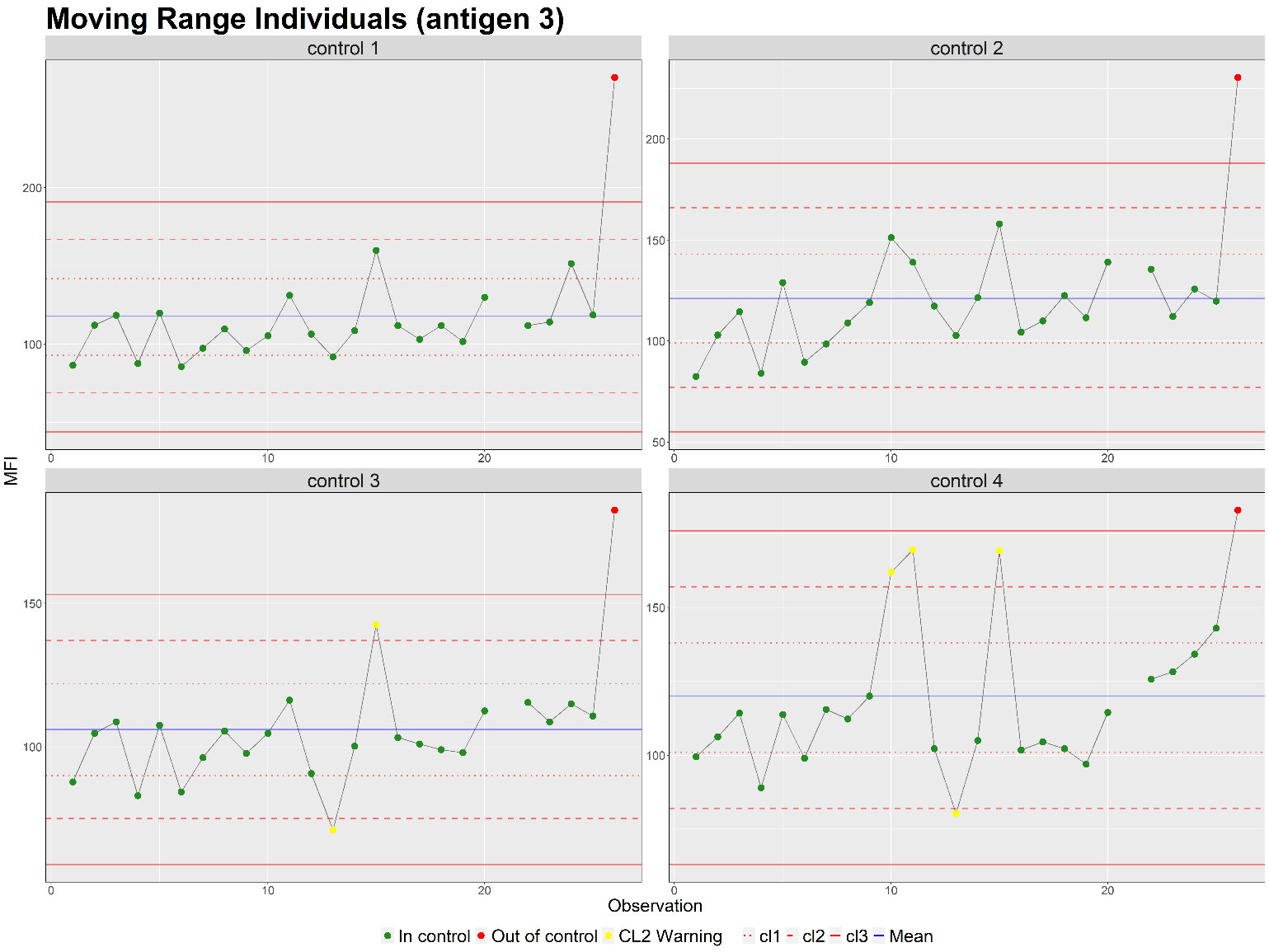


**Supplementary Figure 21.** shinyMBA MFI moving range individuals charts for antigen 3. Datapoints falling outside of the 3σ range were flagged as red “Out of control” and those outside of the 2σ range were flagged as yellow “CL2 Warning”.


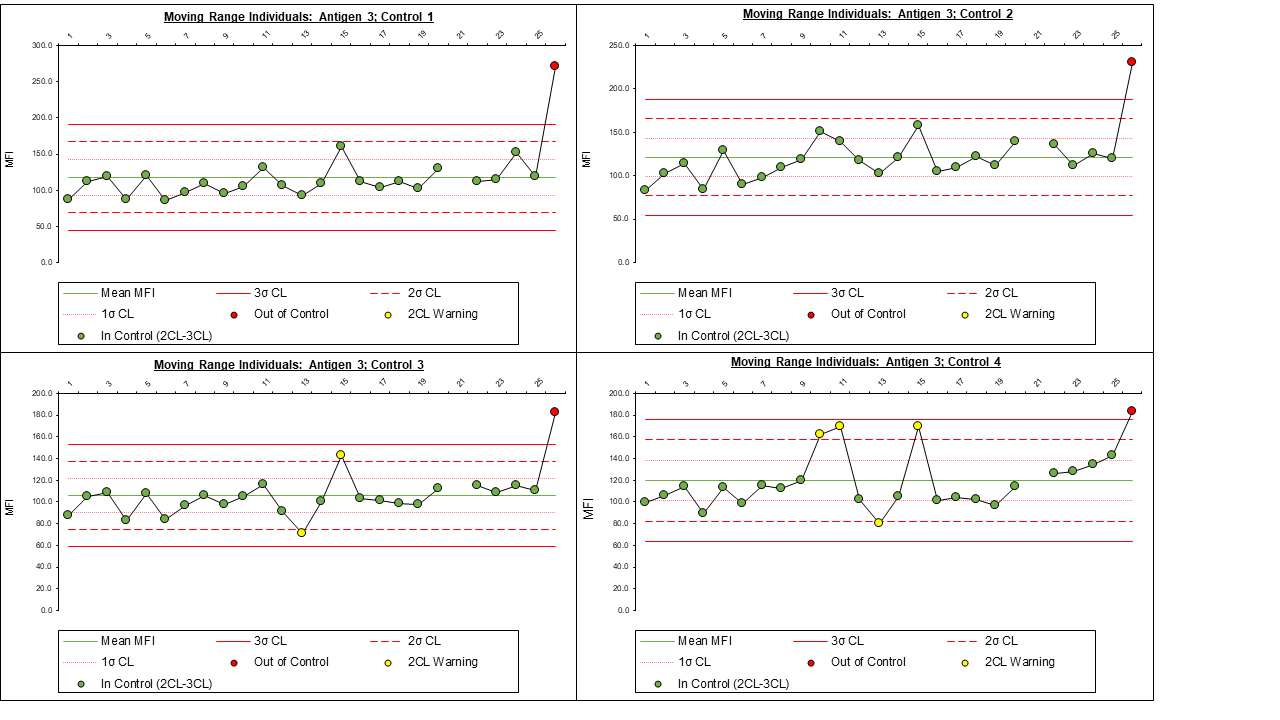


**Supplementary Figure 22.** Excel MFI moving range individuals charts for antigen 3. Datapoints falling outside of the 3σ range were flagged as red “Out of control” and those outside of the 2σ range were flagged as yellow “CL2 Warning”.


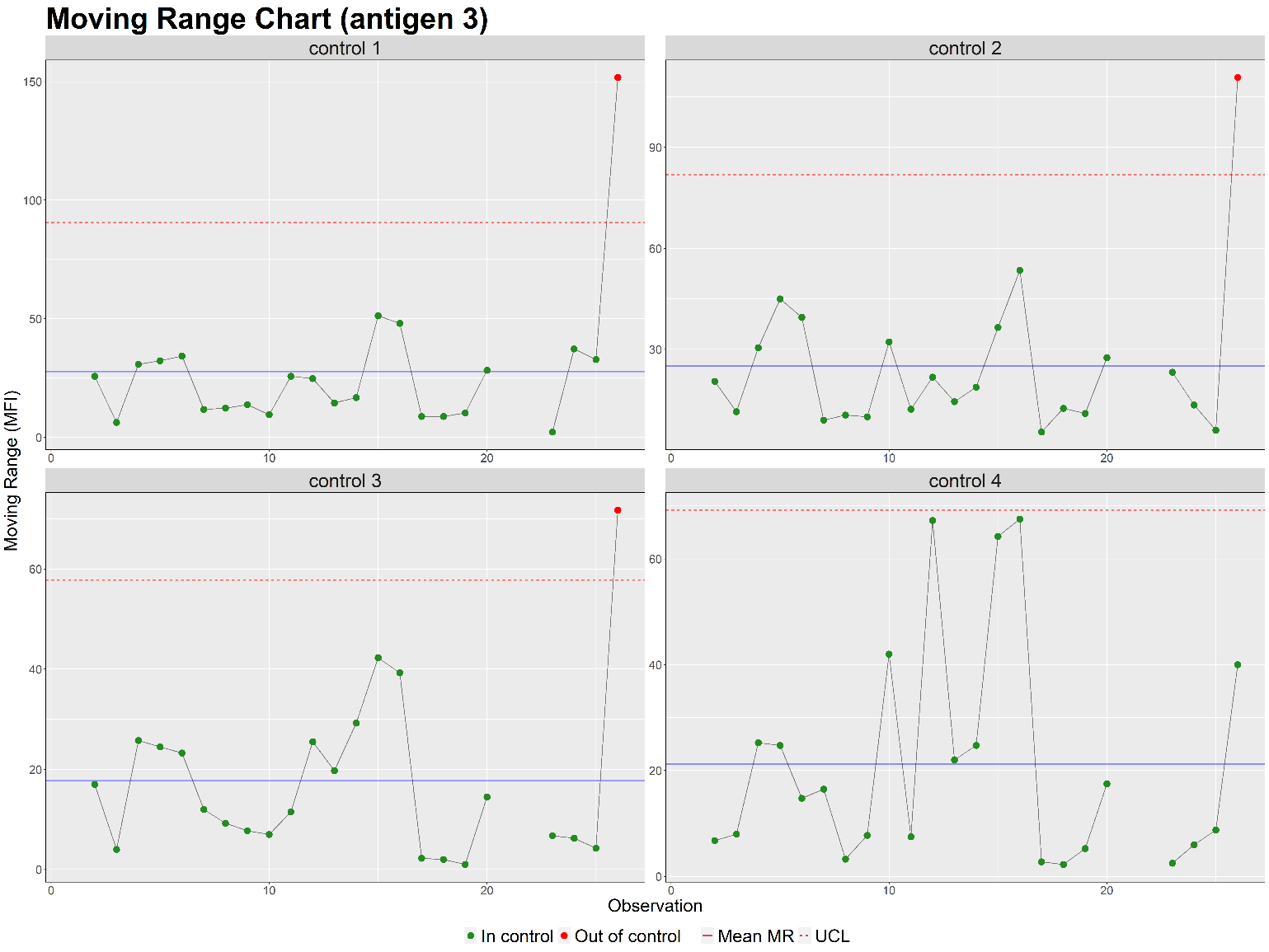


**Supplementary Figure 23.** shinyMBA MFI moving range charts for antigen 3. Datapoints falling outside of the moving range upper confidence limit (UCL) were flagged as red “Out of Control”.


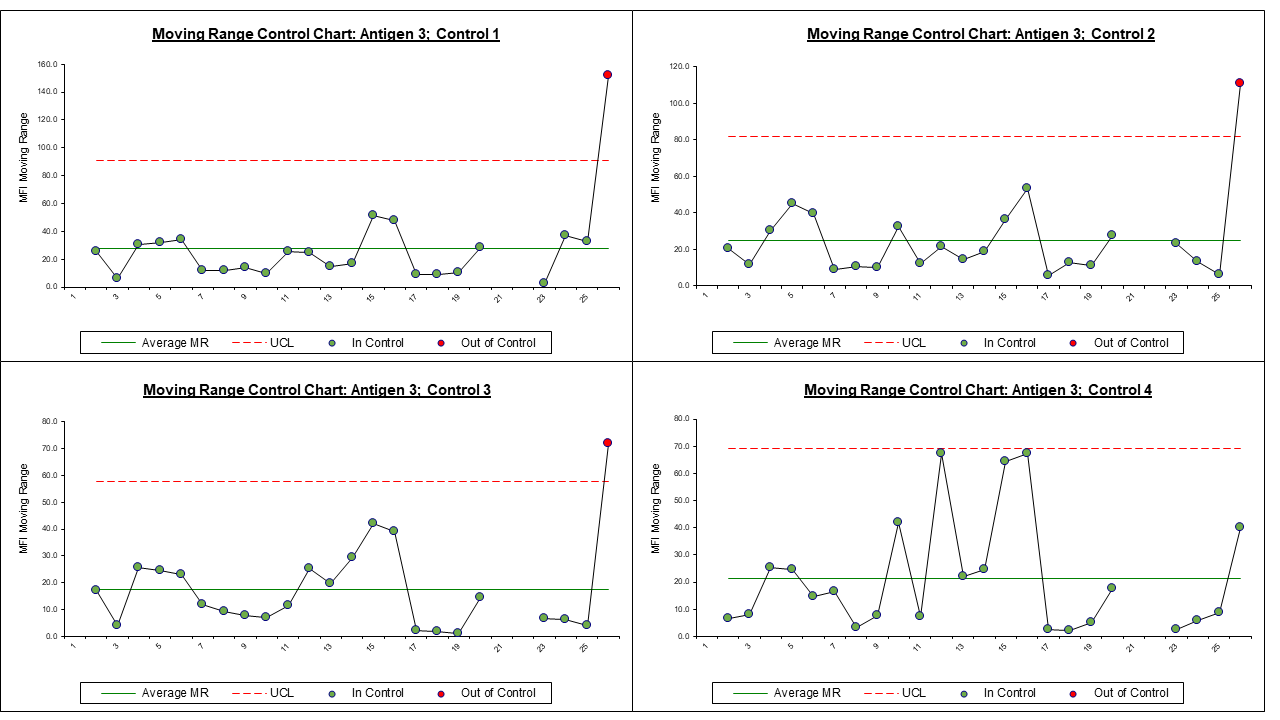


**Supplementary Figure 24.** Excel MFI moving range charts for antigen 3. Datapoints falling outside of the moving range upper confidence limit (UCL) were flagged as red “Out of Control”.


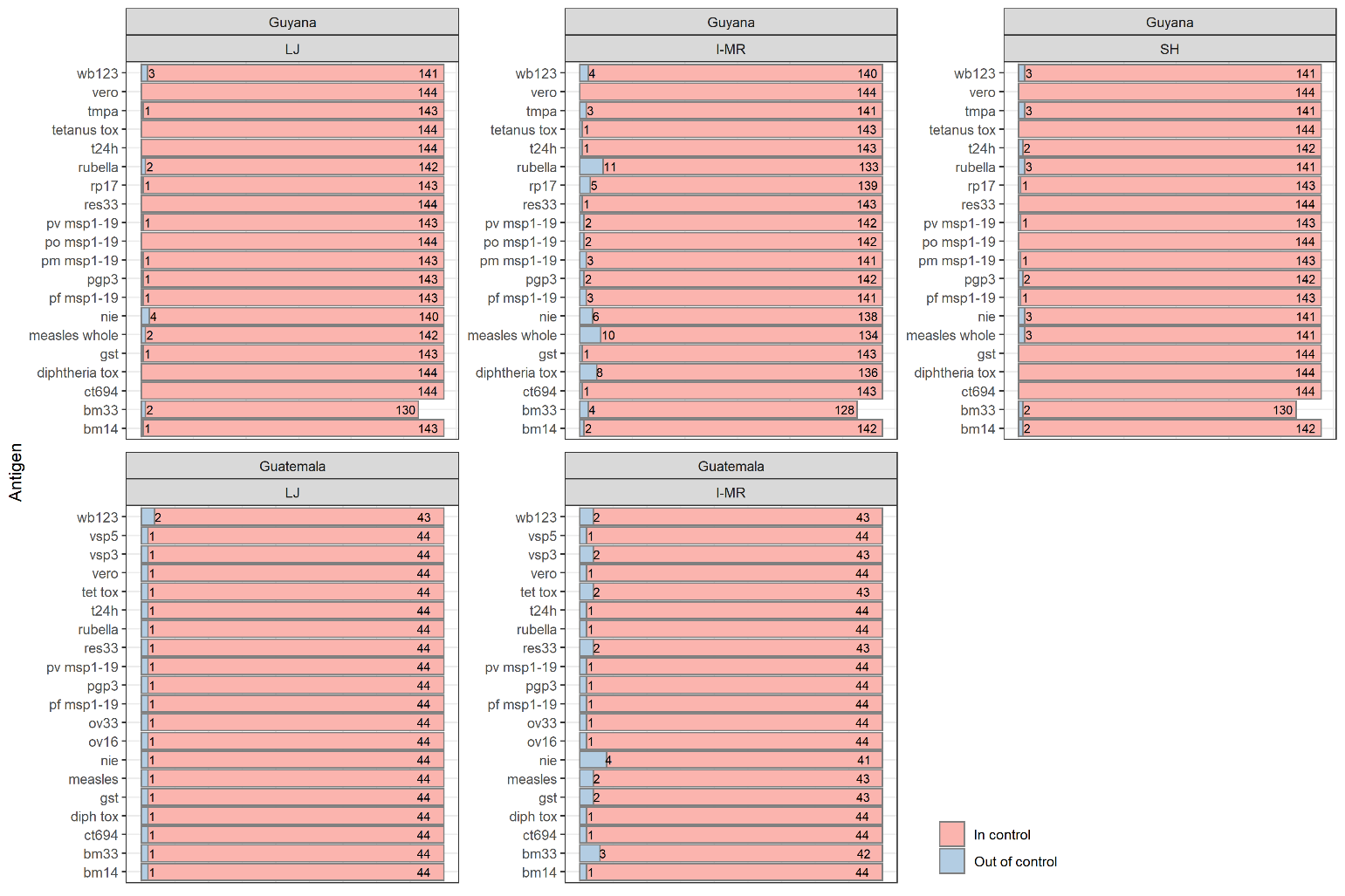


**Supplemental Figure 25.** Control tracking datapoints flagged for each antigen faceted by method. The results of the control tracking analyses were evaluated by antigen for both Guatemala and Guyana studies and compared by stacked bar charts. Three control samples/plate were included multiplied by the number of antigens to yield total data points. Data points outside the 3s range were flagged as “out of control”. Numeric text in the figure denotes the total frequency of “in control” (right) and “out of control” (left) datapoints for each antigen.


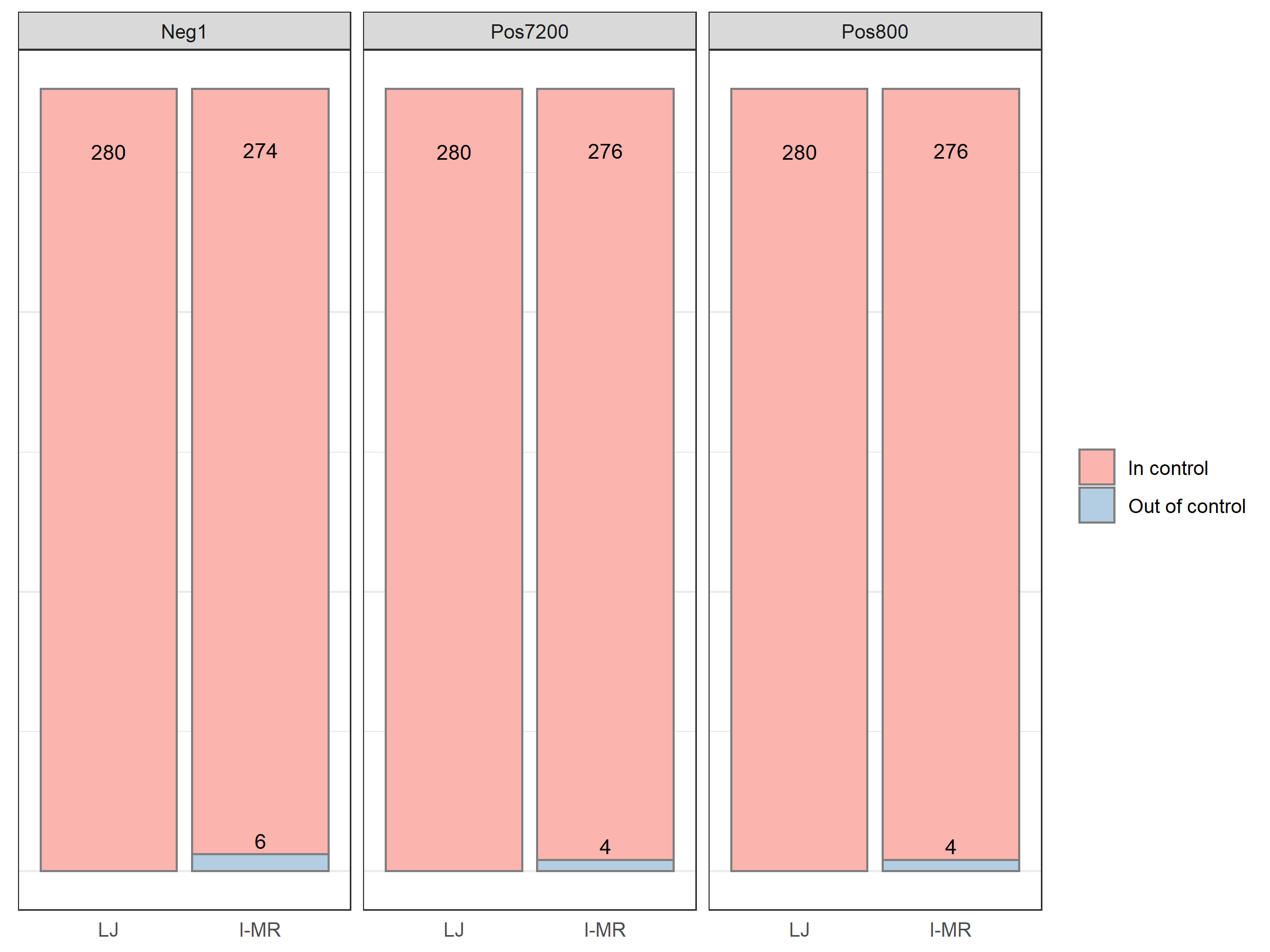


**Supplementary Figure 26.** Levey-Jennings (LJ) and individual moving range (I-MR) QC method comparisons for each control in the Guatemala study with plate 1 outlier removed. For the purposes of the app demonstration, datapoints falling outside of the 3σ control chart range were defined as “out of control” for both QC methods. Numeric text in the figure denotes the total frequency of “in control” (top) and “out of control” (bottom) datapoints for each QC method.


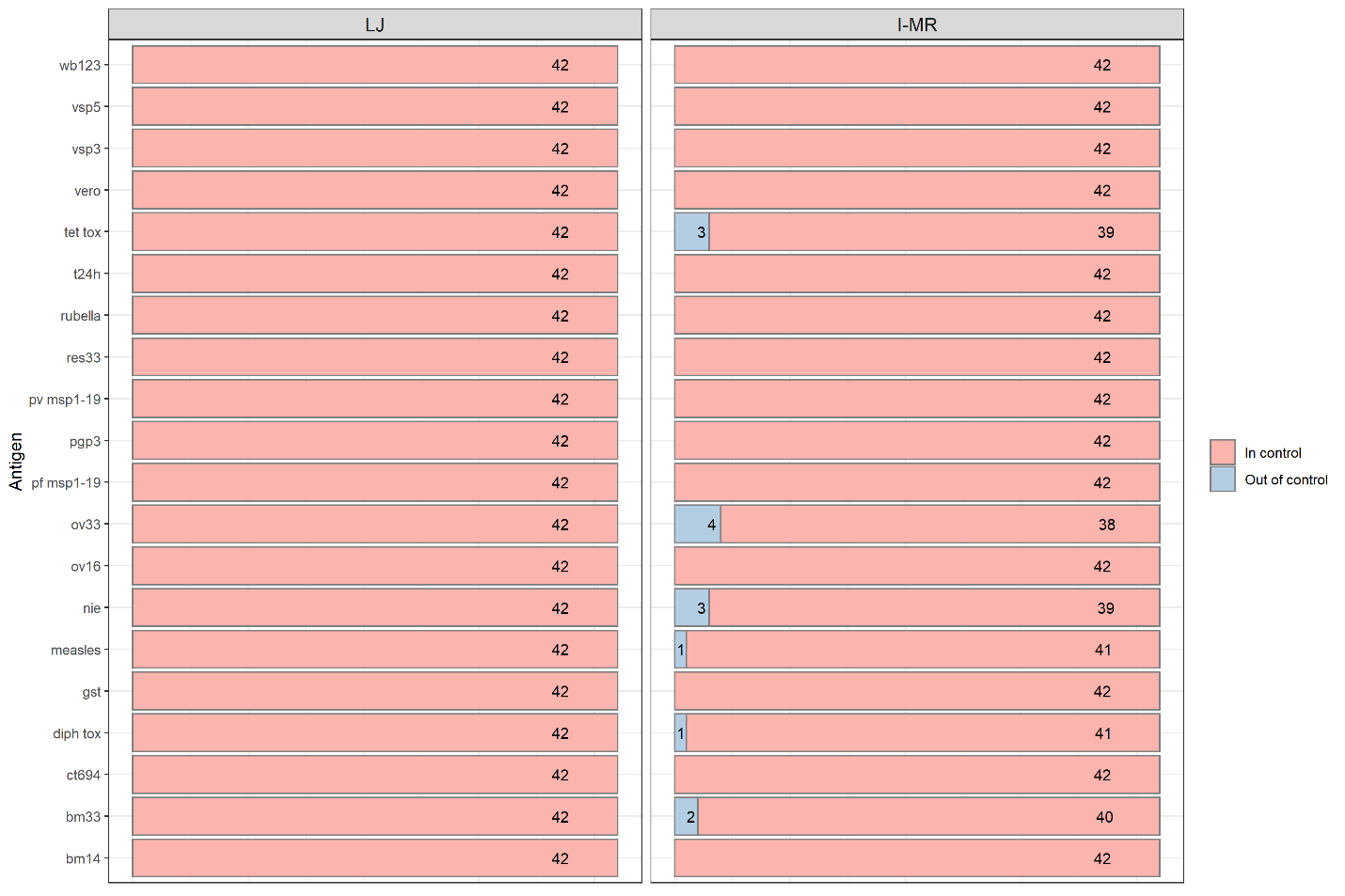


**Supplementary Figure 27**. Levey-Jennings (LJ) and individual moving range (I-MR) QC method comparisons for each antigen in the Guatemala study with plate 1 outlier removed. Datapoints falling outside of the 3σ control chart range were defined as “out of control” for both QC methods. Numeric text in the figure denotes the total frequency of “in control” (left) and “out of control” (right) datapoints for each antigen.
